# Metabolic Phenotypes, Genotypes, and Gut Microbiome Signatures in Obesity: Implications for Precision Nutrition Strategies in Type 2 Diabetes Prevention

**DOI:** 10.1093/nutrit/nuaf088

**Published:** 2025-12-09

**Authors:** Art Muijsenberg, Emanuel E Canfora, Ellen E Blaak

**Affiliations:** Department of Human Biology, NUTRIM Institute of Nutrition and Translational Research in Metabolism, Maastricht University Medical Centre, Maastricht 6229 ER, The Netherlands; Department of Human Biology, NUTRIM Institute of Nutrition and Translational Research in Metabolism, Maastricht University Medical Centre, Maastricht 6229 ER, The Netherlands; Department of Human Biology, NUTRIM Institute of Nutrition and Translational Research in Metabolism, Maastricht University Medical Centre, Maastricht 6229 ER, The Netherlands

**Keywords:** precision nutrition, obesity, T2D, metabotype, glucose metabolism, microbiome, genotype

## Abstract

The worldwide prevalence of overweight and obesity has increased rapidly in the last decades. This rise has led to a surge in comorbidities such as type 2 diabetes (T2D), cardiometabolic diseases, and mental health issues. While general population–based nutrition guidelines have proven effective in reducing T2D incidence by 50%, a significant 30% of participants do not respond to these interventions. Precision nutrition (PN), tailored towards the metabolic phenotype (metabotype), genotype, or microbial characteristics, has shown promise in improving blood glucose control and cardiometabolic health compared with standard nutritional guidelines. This scoping review aims to discuss advancements in PN over the past decade, focusing on heterogeneity in response to dietary interventions aiming to prevent T2D and related cardiometabolic disease in overweight and obese individuals. A semi-systematic PubMed search with defined criteria was conducted to identify precision nutrition (PN) randomized clinical trials and related post-hoc analyses reporting cardiometabolic health outcomes. Numerous studies have shown actionable diet–host interactions, with intervention stratification based on genotype, gut microbiome, metabolome, lipidome, fasting glucose and insulin, postprandial glycemic response, tissue-specific insulin resistance, or combinations thereof. Many of these metabotypes, genotypes, and microbial signatures allow for accurate cardiometabolic outcome predictions and are actionable targets for future PN research. More recently, machine-learning methodologies in the form of postprandial response prediction models have increasingly been used in PN research. However, prospective evidence on effective PN strategies that may prevent the onset of T2D is currently limited. A mechanistic understanding of response and nonresponse in cardiometabolic outcome improvement is crucial in the development of novel phenotyping methodologies and prediction models in PN. These advancements could lead to more inclusive and effective PN strategies to prevent T2D and related conditions.

## Graphical abstract

**Figure:**
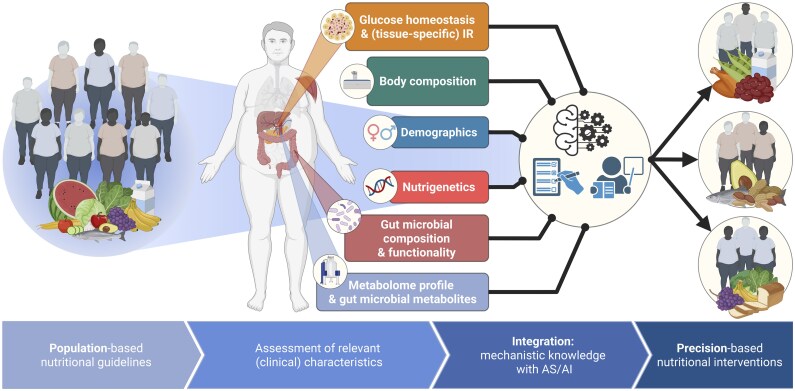

## INTRODUCTION

The worldwide prevalence of overweight and obesity is increasing at an alarming rate, with over 43% of the adult population classified as overweight (body mass index [BMI] >25 kg/m^2^), of whom 16% can be classified as obese (BMI >30 kg/m^2^).^1^^,^^2^ As a result, the prevalence of comorbidities such as type 2 diabetes (T2D), cardiometabolic diseases, as well as impaired mental health and depression has grown dramatically over the past decades. A causative factor for this major global health and socioeconomic problem can be found in our current obesogenic environment: easy access to energy-dense foods and a sedentary lifestyle.^1^

The development of T2D in individuals with overweight and obesity typically occurs gradually over several decades, with variations in the development of T2D and other metabolic syndrome (MetS) hallmarks among those at risk.^3^ Some individuals with obesity appear to be relatively protected against disturbances in metabolic health, a phenomenon called “metabolically healthy obesity” (MHO), which is potentially also a transient state towards “metabolically unhealthy obesity” (MUO).^4^ This can be further explained by the notion that the absolute amount of adipose tissue (AT) is a lesser predictor of metabolic health compared with body fat distribution, characterized by either predominant abdominal (upper body; associated with increased risk) or gluteofemoral (lower body; associated with decreased risk) AT depots.^4^ Furthermore, AT function rather than fat mass per se may determine cardiometabolic risk. Adipose tissue dysfunction is characterized by a reduced lipid buffering capacity as well as inflammation (ie, infiltration of immune cells).^5–7^ Specifically, the role of AT lipid overflow induced by high dietary fat intake as well as an altered secretion of inflammatory factors, resulting in lipotoxicity and low-grade systemic inflammation and subsequent development of peripheral insulin resistance (IR) and T2D, is well recognized.^8^^,^^9^

Current general population–based guidelines for a healthy diet have proven effective in reducing T2D incidence in individuals with prediabetes by up to 50% when individuals were supported and guided to incorporate these guidelines into their daily life.^10^ Notwithstanding, a significant 30% of participants within these programs do not respond or adhere to such interventions.^10^^,^^11^ Precision nutrition (PN), as part of personalized nutrition, has emerged as a promising additional approach to increase intervention efficacy and adherence. Precision nutrition seeks to develop effective nutritional advisory approaches on the basis of a combination of the genetic, environmental, personal, and lifestyle characteristics.^12^^,^^13^ Precision nutrition also includes stratified or targeted nutrition, which is a tailored approach that groups individuals with shared characteristics to create specific advice or solutions for each group. In this field, groups of individuals with similar metabolic characteristics are typically considered to share the same “metabolic phenotype”, or Metabotype. Additionally, PN approaches may take into account similarities in factors such as fecal microbial composition and genetic profiles—extending beyond metabotypes to include microbial phenotypes, enterotypes, and genotypes.

Over the past decade, the field of PN has progressed immensely, with the vast majority of published studies and literature emerging during this time. Yet, the concept of providing personalized advice based on a subgroup’s or individual’s genetic, environmental, or lifestyle characteristics is not new. For example, in cases of celiac disease and phenylketonuria, patients have been advised for approximately half a century to refrain from specific foods, in order to prevent negative consequences to their phenotypes’ interaction with consumed gluten or phenylalanine, respectively.^14^^,^^15^ While the aforementioned examples focus on very specific (genetic) individual characteristics, the basis for stratification of precision-based nutritional interventions can also be much more holistic. Most often, a combination of characteristics that define a phenotype is used—for example, relating to an individual’s genetics; gut microbiome; metabolome; lipidome; postprandial glycemic response; glucose, insulin, and lipid metabolism; and tissue-specific IR. Additionally, researchers may also choose to stratify intervention groups based on the number or severity of MetS risk factors present in an individual.^12^

The more recent progress in this field has been driven by the use of big data and artificial intelligence (AI) algorithms, which are able to recognize patterns in the complex etiology of T2D and cardiometabolic diseases. A pioneer study conducted by Zeevi et al^16^ demonstrated the feasibility of personalizing glycemic responses using a machine-learning (ML) algorithm that incorporates microbiota composition along with lifestyle and metabolic data. Subsequently, Berry et al^17^ showed in The personalized responses to dietary composition clinical trial (PREDICT) that prediction of postprandial glucose and triacylglycerol (TAG) responses after a glucose load or a mixed meal was feasible using ML models. However, despite these ground-breaking studies, a noticeable gap exists in our understanding of the interplay between genetic predispositions, environmental factors, and individual metabolic responses to dietary interventions. Only a couple of prospective studies have since indicated that PN interventions, tailored to an individual’s predicted glycemic response^18^ or tissue-specific IR phenotype,^19^ yield superior outcomes in blood glucose control and insulin sensitivity, and potentially enhance intervention adherence compared with population-based nutritional guidelines.

The aim of this scoping review is to consolidate advancements in PN over the past decade, focusing on the heterogeneity in responses to dietary interventions and the identification of pretreatment predictive biomarkers in individuals with overweight and obesity. By exploring gaps in intervention efficacy, including identification of responders and nonresponders, this review seeks to provide insights into innovative nutritional strategies that hold promise for more effective prevention of T2D, MetS, and associated cardiometabolic disorders. Alongside an overview of notable studies, their efficacy, and the rationale for stratified or individualized nutritional advice, we highlight examples of how precise measurements of features in the prediabetic state can be integrated into comprehensive PN strategies, addressing a wider spectrum of individuals at risk.

## METHODS

For the present scoping review, a semi-systematic PubMed search was conducted to find relevant literature. The exact search terms can be found in Table S1. EndNote 21 (Clarivate, Philadelphia, PA, USA) was used to manage the identified literature. Only clinical and randomized controlled trials (RCTs) in adult humans, published between January 2014 and 2024, and written in English were included. Studies were deemed relevant when the main outcome measure was related to cardiometabolic health (eg, glucose homeostasis, IR, body weight [BW], blood lipids). In addition, participants should have been stratified (prospectively or post hoc) by group or on an individual level on the basis of their (degree of) glucose homeostatic disturbances, adiposity, genetics, microbiome, metabolome, inflammatory status, ethnicity, or a combination thereof. Further details on inclusion and exclusion criteria can be found in Table S2. Titles and abstracts of identified articles were screened, and if deemed relevant for the present work, the full text was studied in detail. Several additional relevant studies were identified from the included articles’ reference lists. Included studies were categorized into dedicated sections on glucose homeostasis, (nutri)genetics, gut microbiome, metabolome, and (postprandial) response prediction models. A PRISMA (Preferred Reporting Items for Systematic Reviews and Meta-Analyses) diagram, displaying included studies per section, is shown in Figure S1.

## PARAMETERS OF GLUCOSE HOMEOSTASIS AND INSULIN RESISTANCE AS DETERMINANTS OF INTERVENTION OUTCOME

Stratification of intervention participants based on their IR status or degree of MetS hallmarks may be an interesting avenue to optimize intervention response. Our search and selection revealed 10 post hoc analyses, 1 prospective study, and 1 acute study that focused on parameters of glucose and insulin metabolism in their stratification of participants (see Table 1).^19–30^ In addition, 21 relevant studies (ie, supporting literature) were included from their reference lists via handsearching.

**Table 1.** Overview of Dietary Interventions That Incorporated Prospective and Post Hoc Stratification Based on Parameters of Glucose Homeostasis and Insulin Resistance

Metabotyping/stratification	Study design	Participants	Diet/treatment	Outcome	**Reference**
Tissue-specific IR	Prospective 12-wk, parallel-design RCT, multicenter	242 participants with either isolated MIR or LIR, BMI 25-40 kg/m^2^, aged 40-75 y	PhenoDiet Group A (LIR + LFHP and MIR + HMUFA) vs PhenoDiet Group B (LIR + HMUFA and MIR + LFHP)	Following intervention, Matsuda index (postprandial glucose tolerance) significantly improved by 20% in Group B, compared with only 5% in Group A. Other secondary outcomes (fasting and 2-h insulin levels, insulin sensitivity, TAG concentrations, CRP levels, and body composition) significantly improved in Group B compared with Group A. Improvement in primary outcome measure (Disposition Index) was not statistically significant. Cardiometabolic improvements were achieved, without weight loss and despite 76% of participants having NGT at baseline.	PERSON^19^
IR status	12-wk, parallel-design RCT, multicenter, post hoc stratification analysis	417 participants with MetS, BMI 20-40 kg/m^2^, and aged 35-70 y	4 isocaloric diets of varying FA quantity and quality	Following stratification of participants by IR status, those in the highest HOMA-IR tertile (>2.93) showed greater reductions in fasting insulin and HOMA-IR on HMUFA and both LFHCC diets than the high-SFA control diet, indicating that MetS and IR strongly influence responses to dietary fat quality.	LIPGENE^20^
Normoglycemic vs prediabetic	Post hoc stratification of data from 3 parallel 10–26-wk RCTs with similar diets	1168 participants with OW/OB, aged 18-65 y	Low GL (ie, LFHCC, high fiber or NDD) vs high(er) GL control (ie, Western or ADD).	Participants from the DiOGenes and NUGENOB cohorts with prediabetes (FPG 5.6–6.9 mmol/L) and OW/OB were up to 4 times more prone to weight (re)gain on high-GL diets but achieved significant weight loss on low-GL diets. In the SHOPUS cohort, weight-loss predictions showed 3 kg greater loss per mmol/L increase in FPG and 0.039 kg per pmol/L decrease in FI, favoring the NND over ADD.	DiOGenes, NUGENOB, and SHOPUS^21^^,^^22^
FPG status (normal vs impaired)	2-y, parallel-design RCT, post hoc stratification analysis	180 participants with IGT (2-h PG 7.8-11.1 mmol/L), aged >18 y	Twice-daily 7.5 g insoluble cereal oat fiber supplement or placebo	Stratification by FPG status showed that the IFG group in the fiber treatment arm had significant improvements in 2-h glucose, HbA1c, and gamma-glutamyl transferase compared with the placebo. Post hoc analysis by obesity status indicated that prediabetic state was a better predictor of treatment outcome than body weight. A dose–response relationship between dietary fiber intake and 2-h glucose change plateaued at ∼25 g/d.	OptiFit^23–27^
Normoglycemic vs. prediabetic cluster based on MMT	Baseline data parallel-design RCT, multicenter	155 participants with ≥2 MetS traits	Mixed-meal test with regular foods to assess postprandial glycemic response	Two clusters were identified: Cluster A (“normo-glycemic”) showed lower HbA1c, waist circumference, and insulin sensitivity, while Cluster B (“[pre-]diabetic”) had poorer glycemic control and higher waist circumference. Gut microbiota (eg, *Clostridium sensu stricto 1* and *Blautia*) differed between clusters.	MEDGI-carb^30^
Male vs female	12-wk, parallel-design RCT, multicenter, post hoc stratification analysis	156 participants with ≥2 MetS traits	Low-GI diet vs high-GI diet, matched for carbohydrate (270 g/d) and fiber (35 g/d)	Women on the high-GI diet had significantly higher average 8-h plasma glucose levels after standardized meals compared with those on the low-GI diet (23% higher on day 1, rising to 37% after 12 wk). No significant differences were observed in men. A significant interaction between sex and diet was found, indicating women were more sensitive to higher-GI diets/meals.	MEDGI-carb^28^
IR status	Acute randomized, controlled, crossover design, post hoc stratification analysis	34 participants, BMI 18.5-30 kg/m^2^, aged 50-80 y	Test foods with varying macronutrient composition: high PRO vs high CHO vs OGTT	High-PRO products significantly reduced glucose peak concentrations and iAUC compared with high-CHO products, with no differences in insulin response. Fasting glucose was positively correlated with glucose iAUC for the high-PRO product, while fasting insulin and HOMA-IR were associated with insulin iAUC for both products. Regression models showed both test foods reduced glucose and insulin iAUC compared with OGTT. Results only showed an association of fasting insulin and HOMA-IR with insulin iAUC but not with glucose iAUC to test foods.	Galarregui et al^29^

Abbreviations: ADD, average Danish diet; BMI, body mass index; CHO, carbohydrate; CRP, C-reactive protein; DiOGenes, Diet, Obesity, and Genes; FA, fatty acid; FI, fasting insulin; FPG, fasting plasma glucose; GI, glycemic index; GL, glycemic load; HbA1c, glycated hemoglobin; HOMA-IR, homeostatic model assessment of insulin resistance; HMUFA, diet high in monounsaturated fatty acids; iAUC, incremental area under the curve; IGT, impaired glucose tolerance; IR, insulin resistance; LFHP, low-fat, high-protein, high-fiber diet; LFHCC, low-fat, high-complex-carbohydrate diet; LIR, liver insulin resistance; MEDGI-carb, Mediterranean Diet, Glycaemic Index, and Carbohydrate Intervention Trial; MetS, metabolic syndrome; MIR, muscle insulin resistance; MMT, mixed-meal test; NDD, New Nordic diet; NGT, normal glucose tolerance; NUGENOB, Nutrient-Gene Interactions in Human Obesity: Implications for Dietary Guidelines; OB, obesity; OGTT, oral-glucose-tolerance test; OW, overweight; PERSON, the PERSonalized glucose Optimization through Nutritional intervention randomised trial; PG, plasma glucose; PRO, protein; RCT, randomized controlled trial; SFA, saturated fatty acids; SHOPUS, Study on High-Protein intake in Overweight Subjects in the US; TAG, triacylglycerol.

### Post Hoc Analysis to Identify Parameters of Glucose and Insulin Metabolism That May Influence Intervention Outcome

One such example is a post hoc analysis performed within the scope of the large Diet, genomics and the metabolic syndrome: an integrated nutrition, agro-food, social and economic analysis (LIPGENE) dietary intervention study, in which participants with MetS were randomized to 1 of 4 diets varying in dietary fat content and quality for a 12-week parallel isocaloric dietary intervention study.^20^^,^^31^ Overall, there were no differential effects of the diets on the primary outcome of insulin sensitivity, estimated by intravenous glucose-tolerance test.^31^ However, after stratifying participants by Homeostatic Model Assessment for Insulin Resistance (HOMA-IR) tertiles, those in the highest tertile (>2.93) showed significantly greater reductions in fasting insulin and HOMA-IR on the high–mono-unsaturated fatty acid (HMUFA) and both low-fat, high-complex-carbohydrate (LFHCC) diets compared with the high-saturated-fatty-acid (high-SFA) reference diet.^20^^,^^31^ Thus, individuals with MetS and IR seemed more responsive to metabolic health improvements from SFA substitution, indicating that these traits may greatly affect responses to quantity and quality of dietary fat intake. These outcomes provide evidence for the value of targeted dietary macronutrient modulations.

In addition to IR status, fasting plasma glucose (FPG) and fasting insulin (FI) levels also appear to be good predictors of weight (re)gain following hypocaloric or ad libitum diets.^21^ Namely, Hjorth et al^21^ conducted a re-analysis of 2 large multicenter RCTs—the Diet, Obesity and Genes (DiOGenes)^32^^,^^33^ and Nutrient-gene interactions in human obesity: implications for dietary guidelines (NUGENOB)^34^ studies—along with the Danish Study on High-Protein intake in Overweight Subjects in the US (SHOPUS)^35^ study. Despite variations in study design, all prescribed diets were either low in glycemic load (GL), LFHCC, and/or high in fiber compared with a high(er) GL control.^21^ They found that participants with higher FPG and conversely lower FI (re)gained significantly more weight on a high-GL diet compared with those with lower FPG or higher FI, while no differences were apparent between diets in individuals with normal glucose control.^21^ Hjorth et al^21^ concluded that this re-analysis revealed that prediabetic (FPG: 5.6–6.9 mmol/L) individuals with overweight or obesity are up to 4 times more susceptible to weight (re)gain on diets high in GL, while significant weight loss can be achieved on low-GL diets.^33–35^ Subsequently, weight-loss predictions were modeled in the SHOPUS study cohort, based on the previously identified diet baseline FPG, FI, and diet interactions.^22^^,^^35^ Results from this analysis indicated that, for each millimolar increase in baseline FPG, there was a predicted difference of 3 kg more weight loss between diets, comparing the interventions’ New Nordic high-fiber and whole-grain diet (NND) with the average Danish (Western) diet (ADD) control.^22^ In addition, for each picomolar decrease in FI levels, predicted weight loss increased by 0.039 kg, comparing the NND and ADD.^22^

These beneficial effects of increased dietary fiber consumption on several health outcomes, in relation to the participants’ baseline FPG levels, have also been shown in the Optimal Fiber Trial (OptiFit).^23^ In this RCT, a total of 180 participants were recruited with impaired glucose tolerance (IGT; 2-h plasma glucose between 7.8 and 11.1 mmol/L), and randomized into an intervention group receiving a twice-daily 7.5-g insoluble cereal oat fiber supplement or placebo for a period of 2 years. Overall, no differences were observed in diabetes incidence or 2-hour glucose levels between intervention groups.^23^ However, upon re-analysis following stratification of participants based on FPG levels (normal FPG [NFG] and impaired FPG [IFG]), the IFG group in the fiber treatment arm significantly improved their 2-hour glucose levels, glycated hemoglobin (HbA1c) and gamma-glutamyl transferase levels compared with the group with IFG in the placebo arm, following 1 year of treatment.^24^ Interestingly, following another stratified post hoc analysis by participant obesity status (BMI >30 kg/m^2^), researchers concluded that BW is an inferior predictor of intervention outcome compared with the prediabetic state.^25^ Furthermore, an inverse nonlinear dose–response relationship was observed between dietary fiber intake (habitual and supplement) and change in postprandial 2-hour glucose levels, wherein a plateau phase was reached at approximately 25 g/day total intake.^26^

In a post hoc analysis of the multicenter MEDGI-Carb trial,^36^ participants with 2 or more MetS traits were clustered using data from a baseline standardized mixed-meal test with regular foods using only 4 parameters of glucose homeostasis—namely, fasting glucose levels, glucose decay, sinusoidal amplitude, and frequency.^30^ This resulted in 2 clusters: one was a more “normoglycemic” cluster A, which was inversely associated with HbA1c levels, waist circumference, and increased insulin sensitivity, whereas the “(pre-)diabetic” cluster B had higher HbA1c levels, increased waist circumference, and decreased insulin sensitivity.^30^ Consistent with prior research into glucose control, gut microbial genera, such as *Clostridium sensu stricto 1* or *Blautia*, were found to be associated with clusters A and B, respectively.^30^^,^^37^ As the authors stated, these results could be highly relevant for future work aiming to bridge the gap between clinical and at-home diagnosis of glycemic status and cluster identity.^30^ This model holds promise for application using solely dynamic continuous glucose monitoring data obtained remotely following a standardized meal instead of the standard oral-glucose-tolerance test (OGTT) in a clinical setting.^30^^,^^38^ Additionally, data from the same trial showed that women are more sensitive to high-GI diets compared with men with regard to detrimental metabolic effects.^28^

### A PN Strategy Focused on Tissue-Specific IR Metabotypes

Insulin resistance can develop simultaneously across various tissues in the etiology towards T2D in individuals with overweight and obesity, which is typically a gradual process spanning multiple decades.^3^ However, there are also subgroups that display tissue-specific variability, with either more pronounced liver IR (LIR), skeletal muscle IR (MIR), or a discordance between AT IR and HOMA-IR.^39^^,^^40^ From an OGTT, the muscle insulin sensitivity index (MISI; inverse measure for MIR) and hepatic insulin resistance index (HIRI; measure for LIR) can be determined based on previously validated methods.^41^^,^^42^ As confirmed by other studies in this field, these tissue-specific IR metabotypes may already be present in the overweight state, and the MIR and LIR metabotypes may translate into the prediabetic state as isolated IFG (associated with LIR) or isolated IGT (associated with MIR).^19^^,^^43–45^

In addition to features inherent to glucose homeostasis, the tissue-specific IR phenotypes MIR and LIR also show discordance regarding their metabolome, lipidome, and gut microbiome profiles, as well as markers of (systemic) inflammation.^39^^,^^46–49^ For example, a deeper mechanistic analysis revealed that tissue-specific IR was associated with distinct abdominal subcutaneous AT (ScAT) transcriptome profiles.^46^ In individuals with LIR, the expression of ScAT genes related to extracellular remodeling was upregulated, whereas in those with MIR, expression of genes linked to inflammation was upregulated.^46^ Additionally, systemic low-grade inflammation was specifically associated with MIR but not LIR, suggesting that elevated ScAT inflammatory gene expression may drive systemic inflammation, thereby linking ScAT inflammation to the MIR phenotype.^46^ The prevalence of tissue-specific IR metabotypes also differs between sexes, as, in general, women have a higher prevalence of MIR than LIR, suggesting a differential etiology towards T2D and cardiometabolic disease as compared with males.^5^^,^^19^^,^^50^ This notion is further supported by Trouwborst et al,^50^ who showed that, even though men and women had similar MIR, women displayed lesser degrees of LIR and whole-body IR. Most interestingly, in this post hoc analysis of the European DiOGenes weight-loss–weight-maintenance trial, men lost more weight in an initial weight-loss phase, concomitant with more favorable improvements in HOMA-IR, MIR, and LIR, compared with women.^50^ However, during the 6-month follow-up weight-maintenance phase on an ad libitum diet varying in protein content and glycemic index, on average, men regained weight (1.6 kg) while women lost 0.5 kg.^50^ Following the weight-maintenance phase, women displayed a lower rebound in high-density-lipoprotein (HDL) cholesterol, TAG, and diacylglycerol levels, which were independent of BW, while no significant differences between sexes were found in parameters of glucose homeostasis.^50^

Recent findings indicate that tissue-specific IR metabotypes may vary in tissue fat accumulation, with evidence showing discordant visceral and liver fat phenotypes as well as liver and muscle fat/mass phenotypes.^51–53^ Other findings by Song et al^40^ challenge the accepted paradigm that AT dysfunction and IR always vary concordantly with whole-body (HOMA-IR) and MIR, supporting our concept of multiple tissue metabotypes. Additionally, only approximately one-third of the Dutch population with overweight harbor the MIR/LIR phenotype.^19^ Thus, it is evident that more tissue-specific IR metabotypes can be identified with potential distinct etiologies towards cardiometabolic disease.

### Tissue-Specific IR Metabotypes as Determinants of Intervention Outcome

From the above, it is evident that individuals with MIR and LIR have different cardiometabolic risk profiles and may represent different etiologies towards T2D and cardiometabolic disease. A recent prospective RCT, the PERSON study, conducted by Trouwborst et al,^19^ investigated whether the metabotypes of LIR and MIR may also respond differentially to dietary interventions. Individuals with overweight and obesity were stratified by their degree of tissue-specific IR, based on previously established tertile cutoffs for MISI and HIRI, resulting in the inclusion of only exclusive MIR or LIR participants.^19^^,^^54^^,^^55^ Based on a post hoc analysis in the CORDIOPREV study and further mechanistic studies,^56^ 2 eucaloric diets hypothesized to result in a differential response in MIR and LIR metabotypes were designed within the Dutch dietary guidelines; a low-fat, high-protein, and fiber (LFHP) diet and an HMUFA diet. Interestingly, following a 12-week dietary intervention, participants with LIR responded much better to the HMUFA diet, while individuals with MIR responded much better to the LFHP diet for the outcome variables of whole-body insulin sensitivity (Matsuda index), MISI, C-reactive protein (CRP), as well as fasting plasma TAG. There was no diet–phenotype interaction for the primary outcome measure, Disposition Index.^19^ The improvement in outcomes when individuals received the optimal diet for their metabotype was pronounced and clinically relevant, with a 20% further enhancement of whole-body insulin sensitivity.^19^ For the first time, Trouwborst et al^19^ were able to demonstrate that pronounced improvements in insulin sensitivity and cardiometabolic risk profile could be achieved with a eucaloric macronutrient modulation alone, independent of body-weight loss, and even though the vast majority (76%) of participants were considered to have normal glucose tolerance at baseline.

In line with many other studies, the aforementioned studies emphasize the potential of macronutrient modulations to effectively manage postprandial glucose metabolism, body composition and weight.^29^^,^^57^^,^^58^ In particular, focusing on the modulation of macronutrient intake tailored to either tissue-specific IR parameters (eg, MIR, LIR) or baseline glycemic status (eg, FPG, FI, HOMA-IR), with most of the studies discussed being post hoc analyses (see also Table 1). However, 1 exception within this section is the prospective PERSON study,^19^ highlighting a need for evidence from further well-designed prospective studies within this scope.

## NUTRIENT–GENE INTERACTIONS AND INTERVENTION OUTCOME

Following The Human Genome Project in the early 2000s, culminating in the first-ever sequence of a whole human genome, the field of genomics has increasingly influenced nutritional science. In particular, the study of nutrigenetics postulates that the food and nutrients we consume interact with our genes, resulting in individual responses to diets, potentially leading to discordant etiologies towards cardiometabolic disease.

Within the field of nutrigenetics and PN, study participants are usually stratified on the basis of selected single nucleotide polymorphisms (SNPs), associated with discordant metabolic responses to nutrition intake. Many of the gene–diet interaction studies identified in our search—specifically, 11 studies—are post hoc analyses stratified by genotype and conducted within the framework of larger RCTs. Table 2^59–70^ summarizes findings from these studies, suggesting that individuals carrying specific genetic variants related to glucose homeostasis, lipid metabolism, circadian rhythm, energy balance, and appetite regulation may exhibit differential responses to dietary (macronutrient) modulations. These post hoc analyses suggest that, based on genotypes, modification of their diet may lead to improvements in FPG,^66^^,^^70^ insulin sensitivity,^63^^,^^67^ plasma lipid profiles,^59–61^^,^^63–65^ inflammatory markers,^59^^,^^60^^,^^66^^,^^67^ and body composition,^68^ whereas others may not respond as effectively.^62^^,^^69^ For example, in the CORDIOPREV RCT, a 12-month study involving 507 participants with MetS and established event-free coronary heart disease (aged 20–75 years), adherence to a Mediterranean (MedDiet) vs a low-fat (LF) diet eliminated baseline differences in plasma high-sensitivity CRP (hsCRP) and TAG levels, which were observed following post hoc stratification based on a tumor necrosis factor alpha (TNF-α) SNP.^59^ G/G allele carriers, who initially exhibited higher levels of these markers compared with minor A-allele carriers showed comparable levels only in the MedDiet arm.^59^ Another example comes from the LIPGENE study, a 12-week multicenter RCT involving 442 participants with MetS (BMI: 20–40 kg/m^2^; aged 35–70 years), which demonstrated that baseline plasma levels of total cholesterol (TC), low-density lipoprotein (LDL), and apolipoprotein (Apo) B were significantly higher in carriers of the APOE E4 risk allele compared with carriers of the E2 allele or the E3/E3 genotype.^63^ Plasma SFA (C16:0) levels were linked to IR in E4 carriers. Postintervention, increased plasma n-3 PUFAs correlated with beneficial increases in ApoCII and reductions in detrimental ApoCIII concentrations in E2 carriers.^63^

**Table 2.** Overview of Dietary Interventions That Incorporated (Post Hoc) Stratification Based on SNPs Located on 1 Gene

Gene (SNP)	Study design	Participants	Diet/treatment	Outcome	Reference
*TNFA* (rs1800629)	12-mo, parallel-design RCT, post hoc stratification analysis	507 participants with MetS and established CHD, aged 20-75 y	MedDiet vs LF diet	G/G allele carriers had higher plasma hsCRP, fasting and postprandial TAG levels at baseline compared with minor A-allele carriers. Following MedDiet adherence, baseline differences disappeared.	CORDIOPREV^59^
*CLOCK* (rs4580704)	12-mo, parallel-design RCT, post hoc stratification analysis	897 participants with MetS and established CHD, aged 20-75 y	MedDiet vs LF diet	Following LF diet, C/C allele carriers showed significantly greater decreases in hsCRP and an increase in the ratio of HDL/APO-A1.	CORDIOPREV^60^
*CETP* (rs3764261)	12-mo, parallel-design RCT, post hoc stratification analysis	424 participants with MetS and established CHD, aged 20-75 y	MedDiet vs LF diet	Following MedDiet, a gene–diet interaction was found in carriers of minor TT + TG alleles, who showed higher plasma HDL and lower TAG, compared with major allele homozygotes (GG). No LF gene–diet interactions were found.	CORDIOPREV^61^
*APOE* (rs429358 and rs7412)	6-mo, parallel-design RCT, multicenter, post hoc stratification analysis	1466 participants, no BMI restriction, aged ≥18 y	0-3 increasing levels of treatment personalization	Highest baseline TC concentrations were observed in risk-allele participants. No gene–diet interaction observed following gene-based personalization (level 3).	Food4Me^62^
*APOE* (rs429358 and rs7412)	12-wk, parallel-design RCT, multicenter, post hoc stratification analysis	442 participants with MetS, BMI 20-40 kg/m^2^, and aged 35-70 y	4 isocaloric diets of varying FA quantity and quality	Baseline plasma TC, LDL, ApoB concentrations were higher in risk-allele (E4) carriers compared with E2 and E3/E3 carriers. Higher plasma SFA (C16:0) was associated with IR in E4 carriers. Following intervention, increased plasma n-3 PUFAs were associated with increased in ApoCII (beneficial) and reduced ApoCIII (detrimental) concentrations in E2 carriers.	LIPGENE^63^
*ADRB2* (rs1042713 and rs1042714)	4-mo, parallel-design RCT, post hoc stratification analysis	107 participants with BMI 25-40 kg/m^2^; untreated diabetes not excluded	Hypocaloric LF vs MHP diet (-30E%)	Following an MHP diet, GLY16Gly homozygotes had lower reductions in TC, LDL, and non-HDL levels than Arg16 allele carriers. Similar effects were observed for Gln27Glu SNP and Gly16/Glu27 haplotype. Thus, a hypocaloric LF diet might be more beneficial compared with an MHP diet in Gly16Gly carriers.	Ramos-Lopez et al^64^
*PPARGC1A* (rs8192678 and rs3755863)	4-mo, parallel-design RCT, post hoc stratification analysis	107 participants with BMI 25-40 kg/m^2^; untreated diabetes not excluded	Hypocaloric LF vs MHP diet (-30E%)	Following an LF diet, a gene–diet interaction was observed. Rs8192678 Gly482Gly homozygotes had lower reductions in TC and LDL compared with 482Ser allele carriers. No detrimental gene–diet interaction was observed in the MHP group. Thus, a hypocaloric MHP diet might be more beneficial than an LF diet in Gly482Gly carriers.	Ramos-Lopez et al^65^
*FADS1* (rs174550)	4-wk, parallel-design	59 healthy men with BMI <30 kg/m^2^	Habitual diet, supplemented with LA (PUFA)	Plasma LA proportion increased in both genotype groups following an LA-enriched diet. However, a gene–diet interaction was observed regarding improvements in FPG, wherein CC homozygotes improved while TT homozygotes did not. Regarding hsCRP improvement, a trend towards an opposite effect was observed.	FADSDIET^66^
*FADS1* (rs174550)	8-wk, randomized parallel-design, post hoc stratification analysis	130 healthy men with BMI <32 kg/m^2^	Reduced-fat (habitual) diet, supplemented with LA or ALA (PUFA)	Following the LA-enriched diet, a significant gene–diet interaction was observed regarding improvements in DI_30_ and hsCRP levels in CC genotype participants. No gene–diet interaction in terms of clinical improvement was observed following the ALA-enriched diet.	FADSDIET2^67^
*FTO* (rs9939609)	6-mo, parallel-design RCT, multicenter, post hoc stratification analysis	683 participants with OW or increased WC (>88 cm in F, >102 cm in M), aged ≥18 y	0-3 increasing levels of treatment personalization	Informed FTO risk allele participants (PN, level 3, AT/AA carriers) showed significant adiposity (BW and WC) improvement compared with controls (non-PN, level 0). BW and WC reductions were higher in FTO risk carriers. However, incorporating genotypic traits had no added value over other PN treatments (levels 1 and 2).	Food4Me^68^
*FTO* (rs9939609)	2-y, parallel-design RCT, post hoc stratification analysis	144 healthy participants, BMI <30 kg/m^2^, aged ≥18 y	Hypocaloric (-25E%) diet vs ad libitum control	Primary endpoint was long-term dietary adherence to caloric restriction, which did not display a genotype-by-treatment interaction. A negligible association was observed in secondary outcomes (body composition, aging biomarkers, and eating behavior).	Dorling et al^69^
*INAFM2* (rs67839313)	Retrospective cohort	7175 participants of whom 4202 with T2D and 2973 related (parent or sibling) T2D-free controls; all aged ≥40 y	Stratified by median egg consumption; high, ≥4/wk, vs low, <4/wk	A significant gene–diet interaction was observed among T-allele carriers. Participants with low egg consumption had higher T2D risk than those with high egg consumption. Among non-T2D T-allele carriers, a significantly increased or decreased FPG was associated with low or high egg consumption, respectively. This interaction was only observed in Asian populations.	Wang et al^70^

Abbreviations: ADRB2, beta-2-adrenergic receptor; ALA, alpha-linoleic acid (18:3n-3); APO, apolipoprotein; APOE, apolipoprotein E; BMI, body mass index (kg/m²); BW, body weight; CETP, cholesteryl ester transfer protein; CHD, coronary heart disease; CLOCK, circadian locomotor output cycles; DI30, 0–30 min Disposition Index; E%, percentage of energy; FA, fatty acid; FADS1, fatty acid desaturase 1; FPG, fasting plasma glucose; FTO, fat mass and obesity-associated gene; hsCRP, high-sensitivity C-reactive protein; INAFM2, indoleamine 2,3-dioxygenase activating factor 2; LA, linoleic acid (18:2n-6); LF, low-fat; M, male; MetS, metabolic syndrome; MHP, moderately high-protein; OW, overweight (defined as BMI ≥ 25 kg/m²); PN, precision nutrition (group); PPARGC1A, peroxisome proliferator-activated receptor gamma coactivator 1-alpha; PUFA, polyunsaturated fatty acid; RCT, randomized controlled trial; SNP, single nucleotide polymorphism; T2D, type 2 diabetes; TAG, triacylglycerol; TC, total cholesterol; TNFA, tumor necrosis factor-alpha; WC, waist circumference.

Unfortunately, prospective studies within this scope are very limited; however, 1 exception (also listed in Table 2), is the FADSDIET trial.^66^ In total, 59 normoglycemic men without obesity (BMI <30 kg/m^2^) were included from a previously genotyped larger (*n* = 1337) cross-sectional cohort.^71^^,^^72^ All included participants to the trial were known to be either CC or TT homozygotes of the fatty acid desaturase (*FADS1*) gene.^66^ Participants were stratified according to their genotype before the intervention, and no significant differences were observed in baseline characteristics between genotypes with the exception for age (GG participants were slightly older).^66^ Subsequently, all participants followed the same intervention—namely, their habitual diet supplemented with linoleic acid (LA; 18:2n-6) for 4 weeks.^66^ While the plasma proportion of LA increased in both genotypes, only CC homozygotes improved in their FPG following supplementation.^66^ Conversely, a trend towards an improvement in hsCRP levels was observed in TT homozygotes, while an opposite trend was observed in CC homozygotes.^66^ According to the authors, the latter finding could be attributed to conversion of LA to proinflammatory metabolites (eg, eicosanoids).^66^

Indeed, many gene–diet interactions emerge when stratifying individuals solely by a single SNP in aforementioned post hoc analyses. However, another more recent approach involves conducting genome-wide association analysis (GWAS), and constructing a subsequent genetic risk score (GRS) by combining multiple SNPs associated with a response variable. Often, the GWAS (or similar) analysis is performed in large observational cohorts, whereafter the GRS is validated in a clinical trial. Specifically, our search identified 8 studies, which are summarized in Table 3.^73–80^ These studies identified genotypes hypothesized to exhibit varying responses to diet, impacting energy balance,^73^^,^^74^^,^^76^^,^^77^^,^^79^^,^^80^ carbohydrate digestion,^74^ glucose homeostasis,^75^ inflammation,^76^ and lipid metabolism^77^^,^^78^^,^^80^ in observational cohorts. Subsequently, most of these studies^73^^,^^74^^,^^76^^,^^77^^,^^79^^,^^80^ then aimed to validate these genotypes' responses to dietary changes in prospective cohorts and RCTs, suggesting that some gene–diet interactions can enhance weight loss,^73^^,^^74^^,^^79^ improve body composition,^74^ or affect inflammatory markers.^76^ Conversely, others did not elicit a significant gene–diet response in cardiometabolic risk improvement.^77^^,^^80^

**Table 3.** Overview of Dietary Interventions That Incorporated Stratification Based on SNPs Located on Multiple Genes and/or Combined in a GRS/PRS or Prediction Model

**Genotyping**	Study design	Participants	Diet/treatment	Outcome	Reference
*MTNR1B* (rs10830963) MC4R (rs17782313) FTO (rs9939609)	3–6-wk, prospective parallel-design	167 participants with OW; no inclusive age range reported, mean age 51.9 y	Hypocaloric diet (-600 kcal/d)	MTNR1B CG/GG allele females, especially those who consumed higher habitual protein, showed lower weight loss at first follow-up. Carriers of both FTO and MC4R risk allele combined with the MTNR1B CC allele lost more weight instead of less, compared with none or single carriers.	Goni et al^73^
AMY1-GRS based on 9 SNPs	Post hoc stratification analysis and prospective 2-y, parallel-design randomized trial	32 054 participants in 4 cohorts and 811 participants with BMI ≥25 kg/m^2^	Four hypocaloric diets, varying in energy derived from CHO (low, 35E%; moderate 1, 45E%; moderate 2, 55E%; high, 65E%)	Among women in the cohort studies, high AMY1-GRS was associated with increased adiposity if dietary CHO intake was also high. Conversely, in high AMY1-GRS participants with lower CHO intake, less gains in adiposity were observed. In the RCT, CHO intake significantly modified AMY1-GRS associations with changes in BW and WC. Higher GRS was linked to greater BW and WC reductions on a low-CHO diet, while opposite effects were observed on high-CHO diets.	POUNDS Lost^74^
GRS combining 8 SNPs associated with HOMA-IR change	6-wk parallel design, post hoc stratification analysis	138 participants with BMI 25-40 kg/m^2^, who previously showed reduced TAG levels following n-3 PUFA supplementation	Healthy population-based diet, supplemented with 5 g/d fish oil (n-3 PUFA)	Participants were stratified by improvements/stabilization (low risk) or worsening (high risk) of HOMA-IR following supplementation. GWAS analysis identified 8 SNPs associated with HOMA-IR change (suggestive genome-wide significance level), forming the GRS. Cross-validation showed that the GRS significantly predicted increased HOMA-IR risk, despite only suggestive loci identified in GWAS, suggesting cumulative predictive value. The GRS achieved a predictive accuracy of 0.85 for HOMA-IR in the testing dataset and explained 40% of HOMA-IR change variation.	Franck et al^75^
*FTO* (rs9930501, rs9930506, and rs9932754) *ADRB2* (rs1042713 and rs1042714) Combined PRS	Cross-sectional and prospective 6-mo parallel-design RCT	178 healthy participants in the cross-sectional study, no BMI restriction, aged ≥18 y, and 128 healthy participants in the RCT, BMI ≥23 kg/m^2^, aged ≥18 y	Hypocaloric (300-500 kcal/d) diet, high in protein, vitamin E, and fiber vs healthy population-based control diet (<1500 kcal/d)	Cohort participants were stratified into low-, medium-, and high-risk obesity tertiles, based on PRS computed from risk-allele weighted sum. Of these, the high PRS tertile had a significantly higher odds of obesity. Also, significant effects of ethnicity were observed. Following the RCT, significant reductions in hsCRP levels were found in medium and high PRS tertiles, compared with the control.	Tan et al^76^
GRS for 6 CRFs (BMI, BP, LDL, HDL, TAG, FPG)	WHI sub-studies, both observational and 1-y, parallel-design RCT	9000 female participants in GWAS study for CRFs (BMI, BP, LDL, HDL, TAG, FPG); 5000 female participants included in dietary modulation	LF vs habitual control diet	Of the 6 CRFs, only the 1760-variant GRS for LDL cholesterol predicted associated CRF change. Following the dietary intervention, LDL-GRS explained 3.7% of variance in the LF intervention arm, and was not associated with control arm change. Although LDL-GRS could not predict LF diet response, suggestive associations were observed across treatment arms in relation to CHD and stroke.	WHI^77^
Twenty-two candidate SNPs, among those *ABCA1* (rs2066714) and *APOE* isoforms	5 × 4-wk, randomized cross-over trial design	92 participants with elevated WC and low HDL	5 isocaloric diets; 1, high SFA (cheese); 2, high SFA (butter); 3, high MUFA; high n-6 PUFA; high CHO	Consistently, ABCA1 and APOE isoforms were associated with LDL cholesterol. Several target SNPs displayed significant gene–diet interactions. Interindividual variability in LDL cholesterol and TAG concentrations could be explained by multivariate models by 16.0%-33.6% and 17.5%-32.0%, respectively.	Rajendiran et al^78^
Epigenetics: diet-responsive DNA-methylation sites	4-mo, parallel-design randomized trial	442 participants with BMI 25-40 kg/m^2^ and aged 18-67 y	Hypocaloric (-30E%) LF vs MHP diet	Baseline DNA-methylation was assessed, and analyzed for intervention %BMIL, resulting in 2 weighted methylation sub-scores for LF and (11 CpGs) MHP (15 CpGs) diets, respectively. Subsequently, both sub-scores were combined in a prediction (linear mixed-effects) model, which was able to predict %BMIL and optimal diet for 37.3% of participants who completed the intervention (*n* = 201).	García-Álvarez et al^79^
GRS combining 10 SNPs for HF-response and HCHO-response	12-wk, parallel-design randomized trial	145 participants with BMI 27-47.5 kg/m^2^ and aged 18-75 y	4 Hypocaloric (-50 kcal/d) diet-responder groups: 1, HFr + HFd; 2, HFr + HCHOd; 3, HCHOr + HFd; 4, HCHOr + HCHOd	Combined GRS score for 10 SNPs allowed baseline stratification of fat and carbohydrate responders. Following a 12-wk WL intervention, genotype-concordant (1 and 4) did not lose significantly more weight compared with genotype-discordant diet groups (2 and 3). Researchers concluded that current genotyping capability for HFr and HCHOr does not show clear WL benefits of genotype-concordant diets.	POINTS^80^

Abbreviations: AMY1, salivary α-amylase 1; BMI, body mass index; BP, blood pressure; BW, body weight; CHD, coronary heart disease; CHO, carbohydrate; CpGs, cytosine-phosphate-guanine (DNA-methylation) sites; CRF, cardiometabolic risk factor; E%, percentage of energy; FPG, fasting plasma glucose; FTO, fat mass and obesity–associated gene; GRS, Genetic Risk Score; GWAS, genome-wide association study; HCHO, high-carbohydrate; HCHOd, high-carbohydrate diet; HCHOr, high-carbohydrate responder; HDL, high-density lipoprotein; HF, high-fat; HFd, high-fat diet; HFr, high-fat responder; HOMA-IR, Homeostatic Model Assessment for Insulin Resistance; hsCRP, high-sensitivity C-reactive protein; LDL, low-density lipoprotein; LF, low-fat; MC4R, melanocortin-4 receptor; MHP, moderately high protein; MTNR1B, melatonin receptor 1B; MUFA, monounsaturated fatty acid; OW, overweight; PRS, Polygenic Risk Score; PUFA, polyunsaturated fatty acid; RCT, randomized controlled trial; SFA, saturated fatty acid; SNP, single nucleotide polymorphism; TAG, triacylglycerol; WC, waist circumference; WHI, Women's Health Initiative; WL, weight loss; %BMIL, percentage of BMI loss.

One notable example, is a study by Heianza et al,^74^ wherein a GRS based on 9 SNPs located on the Amylase, alpha 1 (*AMY1)* gene—encoding for salivary amylase—was compiled.^81^ It was hypothesized that a higher AMY1-GRS, reflecting increased salivary amylase activity, leads to greater carbohydrate (CHO) bioavailability and subsequent weight gain with increased CHO consumption.^74^ A meta-analysis of 4 large prospective cohort studies involving 32 054 participants revealed that CHO intake significantly and consistently modulated the relationship between AMY1-GRS and changes in BMI and waist circumference (WC) in women.^74^ Among women, a higher AMY1-GRS was associated with increased adiposity gains with high CHO intake and lesser gains with low CHO intake.^74^ These findings were further validated in the POUNDS Lost trial dataset,^82^ a 2-year RCT involving 811 overweight participants of both sexes, who were randomized to 1 of 4 hypocaloric dietary interventions that varied in energy derived from CHO (low, 35% of energy [E%]; moderate 1, 45E%; moderate 2, 55E%; high, 65E%). Results showed that CHO intake modified AMY1-GRS associations with changes in BW and WC.^74^ Higher GRS was associated with greater reductions in BW and WC on a low-CHO diet, while opposite effects were observed on high-CHO diets, indicating that this genetic variant involved CHO digestion may be of importance in designing future gene-based PN strategies.^74^

### Studies With an Integrative Approach With Regard to Genetics in PN

In gene-based PN interventions, genomic data may be combined with other ’omics, such as metabolomics or other biomarker data, albeit using ML algorithms in order to navigate the increased complexity of data to identify clusters with different gene–diet interactions.^83^ For example, the PREVENTOMICS study by Aldubayan et al^83^^,^^84^ developed an ML algorithm that utilized genetic, microbial, biochemical, nutritional, psychological, and behavioral data. The algorithm classified participants with elevated WC and BMI between 27–40 kg/m^2^, aged 18–65 years, into 5 clusters (CHO, lipid, inflammation, oxidative stress, and microbiota) using 35 SNPs and 51 biomarkers.^83^^,^^84^ Participants were randomly assigned to a PN intervention arm (*n* = 49) or a general healthy diet control (*n* = 51) for 10 weeks.^83^ No significant time-by-group interactions were observed for main outcome change in body fat mass, and both groups showed similar significant improvements in IR and blood lipid profiles. The authors concluded that, despite the highly controlled conditions, isocaloric, non–energy-restricted meal-prep boxes for approximately 60% of food intake and app-based recipes for the rest, genetic and biomarker-based PN did not lead to greater improvements in body composition or cardiometabolic risk compared with a predominantly healthy plant-based diet.^83^ It should be noted that, to the best of our knowledge, the clustering algorithm has not been publicly disclosed.^83^^,^^84^ Additionally, the identified clusters might overlap due to shared biomarkers and SNPs, potentially reducing their distinctiveness and the likelihood of a differential dietary response.^84^ Also, clusters were relatively small (eg, microbiota cluster total *n* = 2), which may limit statistical power.^83^^,^^84^

Another example of an integrative approach with regard to genetics in PN is the Food4Me study, incorporating data on both genotype (SNPs Methylenetetrahydrofolate reductase [*MTHFR*]*, Fat mass and obesity-associated [FTO], Transcription factor 7-like 2 [TCF7L2], Apolipoprotein E epsilon 4 allele [APOE e4]*, and *FADS1*) and phenotype (anthropometrics: weight, BMI, and WC; biomarkers: glucose, TC, carotenes, and n-3 index). This multicenter, proof-of-principle, web-based RCT aimed to assess whether more personalized dietary advice elicits greater improvements in eating behavior (primary outcome) and cardiometabolic risk compared with conventional advice.^85^ Participants (*n* = 1607, aged 18–79 years; mean BMI: 25.5 kg/m^2^) were randomized to 3 levels of PN advice for 6 months—namely, conventional advice, advice based on dietary intake data, advice based on dietary intake and phenotype, and advice based on dietary intake, phenotype, and genotype.^85^ While results indeed showed that the combined PN levels significantly improved Mediterranean diet^86^ and Healthy Eating Index scores^87^ compared with a control, while no additional benefit of phenotype- and genotype-based advice was observed.^87^ Additionally, no significant gene–diet interactions were observed following post hoc stratification by APOE^62^ and FTO^68^ risk alleles (see Table 2).

Despite the aforementioned extensive research conducted on gene–diet interactions concerning metabolic responses, there remains a notable gap in evidence supporting highly effective dietary recommendations based on multiple SNPs or genome-wide analyses, particularly when combined with other biomarkers.^88^ Unfortunately, notwithstanding their difficulty to design and conduct, prospective studies within the field of genotype-based PN are currently lacking.

Additionally, we realize that, regarding the field of genetics, some aspects are covered to a lesser extent within the scope of our present work, which mainly focusses on nutrigenetics (ie, effect of genetic variations on nutrient–gene interactions). For example, while this may still have important implications for PN strategies, the field of nutrigenomics (ie, influence of diet on gene expression, including epigenetics) has not been extensively covered, with the exception of 1 study.^79^

## GUT MICROBIAL COMPOSITION AND FUNCTIONALITY AS A DETERMINANT OF INTERVENTION OUTCOME

There is a growing consensus that the interplay among the composition, diversity, and functionality of the gut microbiota has a profound impact on the host's metabolism, and is believed to significantly influence the etiology of conditions such as obesity and associated metabolic disorders, including T2D.^89^^,^^90^ Our search and selection revealed 5 post hoc analyses (see Table 4)^91–95^ that focus only on parameters of the gut microbiome in their stratification of participants (ie, who did not incorporate prediction models including other factors—which will be discussed in the section entitled “Precision Models in PN”). In addition, 22 relevant studies (ie, supporting literature) were included from their reference lists via handsearching.

**Table 4.** Overview of Dietary Interventions That Incorporated (Post Hoc) Stratification Based on Gut Microbial Composition and Functionality

Metabotyping/stratification	Study design	Participants	Diet/treatment	Outcome	Reference
Baseline P/B ratio	6-mo, parallel-design RCT, post hoc stratification analysis	62 participants with increased WC, BMI 20-40 kg/m^2^, and aged 35-70 y	High-fiber/whole-grain (NND) diet vs ADD control diet, ad libitum consumption	Participants with a higher P/B ratio at baseline lost, on average, 3.15 kg more body fat on the NND diet, compared with those with lower baseline P/B ratios, who showed no difference in fat loss.	Hjorth et al^91^
Baseline P/B ratio and P abundance	6-wk, parallel-design RCT, post hoc stratification analysis	46 participants with OW and aged 30-65 y	WG fiber-rich diet vs RW control	Participants with high baseline P abundance lost 1.80 kg more on the WG diet compared with the RW control, while those with low P abundance remained weight stable on both diets. No group–diet associations were found for satiety, glucose metabolism, or fecal SCFA concentrations. Additionally, participants without detectable fecal P lost weight on the WG diet, but gained weight on the RW diet.	Christensen et al^92^
Baseline IR and P/B ratio	24-wk WM intervention following weight loss (≥8% BW), post hoc stratification analysis	126 participants with BMI 28-40 kg/m^2^, aged 18-60 y	Habitual low fiber diet (ADD), supplemented with protein or placebo	Participants with a high baseline P/B ratio experienced greater weight regain—especially those with combined high P/B ratio, high HOMA-IR, and low fiber intake—regaining an average of 5.5 kg.	Hjorth et al^93^
Sex and BMI	Cross-sectional, RCT baseline data	75 participants (39 men and 36 postmenopausal women) with similar diets, matched by age	No dietary intervention (habitual diet)	Men with a BMI >33 exhibited lower Bacteroides abundance compared with women, with Bacteroides abundance decreasing as BMI increased, while in women, it remained stable across BMI ranges. Additionally, men showed a higher prevalence of *Veillonella* and *Methanobrevibacter* and a lower abundance of *Bilophila*, irrespective of BMI. Microbiota variation accounted for 31.17% of BMI, 29.04% of triglycerides, 33.70% of HDL, 46.86% of LDL, and 28.55% of total cholesterol.	CORDIO-PREV^94^
Responder analyses	10-wk, randomized crossover trial design, post hoc stratification analysis	14 male participants with MetS (BMI >27 kg/m^2^, WC > 102 cm, FPG >6.0 mmol/L)	Crossover fully controlled diets supplemented with RS or NSPs (3 wk/each), followed by a fixed WL diet (3 wk)	Diet explained 10% of microbiota variance, with interindividual variance being higher. The RS diet increased Ruminococcaceae and reduced microbiota diversity, while the NSP diet increased Lachnospiraceae. The WL diet reduced Bifidobacteria. SCFA production correlated with the total 16S rRNA gene signal and propionate levels specifically correlated with Bacteroidetes. Responsiveness to dietary changes was inversely associated with baseline microbiota diversity.	Salonen et al^95^

Abbreviations: ADD, average Danish diet; BMI, body mass index; BW, body weight; FPG, fasting plasma glucose; HDL, high-density lipoprotein; HOMA-IR, Homeostatic Model Assessment for Insulin Resistance; IR, insulin resistance; LDL, low-density lipoprotein; MetS, metabolic syndrome; NND, New Nordic diet; NSP, non-starch polysaccharide; OW, overweight; P, Prevotella; P/B, Prevotella/Bacteroides; RCT, randomized controlled trial; RS, resistant starch; RW, refined wheat; SCFA, short-chain fatty acid; WC, waist circumference; WG, whole grain; WL, weight loss; WM, weight maintenance.

Gut microbiota composition and diversity demonstrates notable interindividual and group differences, including variations across sexes, BMI categories, and associated metabolic profiles.^94–97^ Obesity, IR, and T2D may be characterized by alterations in microbial composition and functionality, including a reduced microbial gene richness and a reduced content of short-chain fatty acids (SCFAs)—in particular, butyrate-producing pathways. The balance between colonic saccharolytic (eg, SCFAs) and proteolytic (eg, branched-chain fatty acids [BCFAs], trimethylamine N-oxide [TMAO]) fermentation may be an especially important determinant of host metabolic health.^96^^,^^98^

### Gut Microbial Composition as a Determinant of Intervention Outcome

Recently, several interesting studies have been published regarding post hoc analysis of targeted interventions looking into microbial composition, diversity, and enterotypes. Of note, Hjorth et al studied whether baseline *Prevotella*-to-*Bacteroides* (P/B) enterotype ratio affected weight loss (maintenance), body composition, and parameters of glucose metabolism.^91–93^ The underlying rationale for this study was that species of the genus *Prevotella* are regarded as important fiber-degrading, beneficial gut microbes, and their baseline abundance is positively associated with cardiometabolic improvements following dietary fiber intake.^99^^,^^100^ In one of these studies, participants were randomized to either an NND high-fiber/whole-grain diet or an ADD control to be consumed ad libitum for 6 months.^91^ Following the intervention, individuals who had a higher P/B ratio at baseline lost, on average, 3.15 kg more body fat on the NDD over the intervention period, and no difference was observed in individuals with lower baseline P/B ratios.^91^ Similar results were found in another study by the same group using slightly different diets over a shorter duration, wherein baseline abundance of *Prevotella* and the P/B ratio showed an inverse correlation with changes in body weight on a high-fiber diet.^92^ In this study, participants with high baseline *Prevotella* abundance lost 1.80 kg more over the 6-week intervention following consumption of a whole-grain fiber-rich diet, compared with the refined-wheat control, whereas participants with low *Prevotella* abundance remained weight-stable on both diets.^92^ However, no group–diet associations in satiety sensations, glucose metabolism, or fecal SCFA concentrations were found. Interestingly, participants without any detectable presence of fecal *Prevotella* (0-P), which was 34% of the study population (larger than the reported reference of 13%–15%), also showed significant weight loss following the whole-grain diet, whereas 0-P participants on the refined-wheat diet actually gained weight.^92^

In a subsequent post hoc analysis, Hjorth et al^93^ further characterized the observed microbiome–diet relationship by incorporating baseline IR (HOMA-IR), and assessed its interaction effects on weight-loss maintenance. Following an initial weight-loss phase (≥8% BW), participants were randomized to 1 of 3 protein supplement or placebo groups for a 24-week weight-maintenance intervention, during which participants consumed their habitual (ADD) diet.^93^ Contrary to previously described studies, all diets were relatively low in fiber. The study revealed that individuals with a high P/B ratio experienced greater weight regain compared with those with low P/B ratios.^93^ Individuals with combined high HOMA-IR, high P/B ratio, and low fiber intake regained, on average, the most weight (5.5 kg) during the weight-maintenance period—indicating that P/B ratio and glucose metabolism might modulate the effects of fiber on energy balance. Taken together, individuals with higher baseline P/B ratios may be more susceptible to lose BW, fat mass, and/or prevent weight regain, yet only on high-fiber diets, particularly when accompanied by a lower HOMA-IR (thus, the product of lower FPG and/or FI).^91–93^^,^^101^

### Gut Microbial Functionality as Determinant of Intervention Outcome

As previously mentioned, in both obesity and IR, microbial dysbiosis commonly occurs, characterized by a reduction in butyrate-producing species and overall low gene richness.^102–106^ The SCFA butyrate serves as an important fuel source for colonocytes, thereby maintaining intestinal barrier function, and may also exert systemic effects such as improving insulin sensitivity and attenuating low-grade inflammation.^96^^,^^107^^,^^108^ Notably, the initial microbial phenotype has been shown to predict dietary fiber intervention outcomes^109^ after fecal microbiota transplantation (FMT),^110^ bariatric surgery,^111^ medication use,^112^^,^^113^ or ingestion of noncaloric sweeteners.^114^^,^^115^ For example, a study performed by Kootte at al^110^ underscores the importance of baseline microbiota composition. Here, it was demonstrated that participants with obesity receiving an FMT from lean volunteers showed significant improvement in insulin sensitivity.^110^ Interestingly, these improvements were only observed in participants at 6 weeks post-FMT in those who exhibited a lower microbial diversity at baseline, and disappeared at 18 weeks post-FMT.^110^ Similar effects were observed in an acute randomized crossover study, wherein Canfora et al^116^ administered single doses of various dietary fiber supplements the day before clinical testing. These fibers were chosen based on prior in vitro experiments indicating more distal saccharolytic fermentation in the colon, hypothesized to result in more pronounced health benefits.^116^ Results showed improvements in postprandial glucose concentrations, increased markers of microbial fermentation (ie, plasma butyrate concentrations and breath hydrogen), energy expenditure, and carbohydrate oxidation the day after the 1-day supplementation, although these were only observed in lean, normoglycemic individuals but not in participants with overweight and prediabetes.^116^ Moreover, these effects appeared to be fiber-specific, manifesting only when administering long-chain inulin combined with resistant starch, whereas the combined β-glucan with resistant starch did not yield similar effects, indicating a complex structure-function relationship of dietary fibers.^116^ Importantly, these findings indicate a lack of response in individuals with prediabetes, aligning with previous observations where a 4-week oral butyrate supplementation altered insulin sensitivity and metabolism in lean individuals but not in those with obesity and IR.^117^ In line with the aforementioned studies, a dietary intervention by Cotillard et al^105^ using a 6-week hypocaloric high-protein diet, followed by a 6-week weight-maintenance diet, demonstrated improvements in adiposity and plasma cholesterol levels in individuals with low microbial gene richness, although showing limited effectiveness in reducing inflammatory markers within this subgroup.

A recent pilot study investigated the fermentation profiles of gut-derived metabolites following the consumption of ^13^C-labeled wheat bran. The study identified distinct fermentation patterns among healthy women (*n* = 6; BMI: 23.2 ± 0.9 kg/m^2^; aged 29.7 ± 1.7 years), and categorized participants as high- or low-methane producers based on their gas excretion kinetics.^118^ High-methane producers exhibited enhanced and prolonged exhalation of ^13^CH_4_, whereas in low-methane producers a higher proportion of ^13^C-butyrate and a lower proportion of ^13^C-acetate in plasma and feces was observed, suggesting potential differences in microbiota functionality.^118^ The study also highlighted the differential kinetics of branched SCFAs (isobutyrate, isovalerate) vs linear SCFAs (acetate, propionate, butyrate, valerate) in plasma, emphasizing the complexity of metabolic responses to dietary fiber.^118^ These findings underscore the potential for using noninvasive volatolomics approaches to characterize individual gut microbiota–host responses. Using improved technologies that allow noninvasive real-time measurements of intestinal gases as a metric for microbial fermentation, this approach has the potential to provide a basis for personalized interventions that improve host metabolic health.^119^^,^^120^ Altogether, these studies indicate that the initial microbial composition and functionality (ie, the degree of saccharolytic fermentation and SCFA production) may be important determinants of dietary intervention outcomes.

## METABOLOME PROFILES AS A DETERMINANT OF METABOTYPES AND INTERVENTION OUTCOMES

Interindividual variability in the metabolome among individuals within the overweight and obese population, particularly those at risk for T2D and cardiometabolic disease, has been extensively studied over the past decades.^121^^,^^122^ Indeed, distinct metabolome features often associated with the gut microbiome or host metabolism already appear to be present in at-risk individuals (long) before the onset of disease.^32^^,^^33^^,^^39^^,^^48^^,^^55^^,^^122^^,^^123^ Therefore, metabolomics—the study and quantification of small molecules involved in nutrient and energy metabolism within the circulation and tissues—is increasingly used in the field of PN. Adding metabolomics to PN can help to understand the complex metabolic processes and potentially stratify intervention participants based on their (microbially derived) metabolome profile.^124^ Metabolomics often uses advanced analytical chemistry techniques such as mass spectrometry and nuclear magnetic resonance spectroscopy.^124^ Our search and selection revealed 6 post hoc analyses and 3 acute studies that focus only on metabolites and metabolomics in their stratification of participants (ie, who did not incorporate prediction models including other factors—which will be discussed in the section entitled “Prediction Models in PN s”; see Table 6^39^^,^^48^^,^^123^^,^^125–130^ for an overview of included studies in this section). In addition, 10 relevant studies (ie, supporting literature) were included from their reference lists via handsearching.

**Table 6.** Overview of Dietary Interventions That Incorporated (Post Hoc) Stratification Based on Metabolome Profiles

Metabotyping/stratification	Study design	Participants	Diet/treatment	Outcome	Reference
MHO vs MUHO	4-mo, parallel-design RCT, combined analyses of separate trial in MHO and MUHO participants	215 participants with either MHO or MUHO (ATP-III) with suboptimal Vit. D levels, BMI ≥30 kg/m^2^, and aged 18-50 y	Vit. D supplementation (4000 IU/d) vs placebo	Vit. D supplementation significantly altered plasma metabolomic profiles in MUHO participants, with changes observed in specific metabolites such as acyl-lysophosphatidylcholines (C16:0, C18:0, C18:1), diacyl-phosphatidylcholines (C32:0, C34:1, C38:3, C38:4), and sphingomyelin (C40:4). Interaction analyses showed differential effects between MHO and MUHO phenotypes, particularly in acyl-lysophosphatidylcholines (C16:0, C16:1) and citrulline. Metabolite changes in the MOHU intervention group correlated with improvements in cardiometabolic biomarkers (LDL-C, AST, ALT, HOMA-IR, insulin, CRP, and HbA1c).	Bagheri et al^123^
LH vs MHO vs MUHO	Acute meal challenge test	30 participants aged 35-70 y; metabolically healthy if <3 MetS hallmarks	Single standardized high-calorie meal (∼1330 kcal), 66 g of FA, 141 g of CHO, 5 g of fiber, and 42 g of PRO	Postprandial 0-120-min glucose and insulin AUC responses were similar between MHO and LH groups and significantly lower than in the MUO group. Minor differences in postprandial AA responses were observed between MHO and MUO participants, while 3 PUFAs (linoleic acid, α-linolenic acid, arachidonic acid) showed smaller changes in serum levels post-meal in MHO individuals compared with MUO. Fasting levels of several BCAAs and SFAs (eg, myristic and palmitic acids) correlated with glucose and insulin AUCs. Notable limitations include small sample size (*n* = 30) and a short postprandial sampling duration (120 min).	Badoud et al^125^
Tissue-specific IR	Acute hyper- insulinemic-euglycemic clamp	94 middle-aged adults with varying degrees of IR (44% with T2D)	No diet/treatment; plasma metabolites were analyzed under basal fasting and during insulin stimulation conditions	Participants were classified based on varying degrees of muscle IR (Rd), determined under fasting and euglycemic hyper-insulinemic clamp conditions. Plasma BCAA levels were strongly associated with IR indices, particularly hepatic, adipose, and whole-body IR, while lower BCAA levels were linked to muscle IR. Plasma BCAA correlated positively with long-chain acylcarnitines, indicating modulation of mitochondrial metabolism.	Sunny et al^126^
Fasting metabolite levels	Randomized crossover meal challenge test, post hoc stratification analysis	19 postmenopausal women with normal FPG and NGT; mean age of 61 ± 4.8 y and BMI of 26.0 ± 2.5 kg/m^2^	Participants consumed meals containing RWB (control), RRB, or WRB, each providing 50 g of available CHO	Postprandial metabolic responses were measured using NMR and targeted LC-MS. RWB produced higher postprandial concentrations of leucine and isoleucine compared to RRB and WRB. Women with higher fasting concentrations of BCAAs and lower sphingomyelins and phosphatidylcholines showed higher postprandial insulin responses, despite similar glucose responses across bread types. These findings suggest that fasting metabolic profiles can predict postprandial insulin demand.	Moazzami et al^127^
Metabolic health status and BMI	Acute HFMM challenge test	1002 participants subdivided in 6 combinations of BW (NW, OW, and OB), further categorized as MH or MUH (≥2 cardiometabolic abnormalities); all aged 20-75 y, established CHD (6-mo event-free)	HFMM (0.7 g fat/kg BW; 12% SFA, 10% PUFA, 43% MUFA, 10% PRO, 25% CHO)	MH individuals exhibited lower postprandial plasma TAG and large TAG-rich lipoprotein TRL-TAG responses compared with MUH individuals, regardless of BW status. The AUC for TAG and large TRLs-TAG was significantly higher in MUH groups. Additionally, MUH individuals showed higher postprandial hsCRP levels across all BW categories.	CORDIOPREV^128^
T2D RE vs non-RE	5-y parallel-design RCT, post hoc stratification analysis	190 participants with newly diagnosed T2D at baseline; all aged 20-75 y, established CHD (5-mo event-free)	LF diet (<30E% total FA, <10% SFA, 12%–14% MUFA, 6%–8% PUFA, 15% PRO, and ≥55% CHO) or MedDiet (≥35E% total FA, <10% SFA, 22% MUFA, 6% PUFA, 15% PRO, ≤50% CHO)	12 Metabolites (lipids, bile acids, amino acid derivatives, and heme derivatives) were significantly elevated in the non-RE group compared with the RE group. The high metabolite score tertile was associated with a higher probability of remission (HR = 2.70). Inclusion of these metabolites improved the predictive power for remission (AUC = 0.72 vs 0.61 for clinical variables alone; sex, age, BMI, HDL, TAG, and statin use). No differential effects between diets were reported.	CORDIOPREV^129^
T2D vs non-T2D	Cross-sectional baseline metabolomics, post hoc stratification analysis	154 men (aged 55-80 y) and women (aged 60-80 y), with T2D and/or 3 ≥ CVD risk factors; 85 T2D and 69 non-T2D	No diet/treatment; urine metabolites were analyzed under basal fasting conditions	Of the 33 significantly different metabolites between groups, the urinary metabolite signature of T2D was characterized by high levels of methylsuccinate, alanine, dimethylglycine, and guanidoacetate, and reduced levels of glutamine, methylguanidine, 3-hydroxymandelate, and hippurate. This multi-metabolite signature achieved an AUC of 96.4% for T2D prevalence. Participants with T2D with a higher FPG also had higher phenylalanine, phenylacetylglutamine, p-cresol, and acetoacetate levels—indicative of alterations in AA, CHO, and microbiota metabolism.	PREDIMED^130^
Tissue-specific IR	Cross-sectional baseline metabolomics, post hoc stratification analysis	640 participants, FPG <6,1 mmol/L, BMI 27–45 kg/m², aged 18-65 y	No diet/treatment; plasma metabolites were analyzed under basal fasting conditions	MISI was associated with higher LPC levels in both sexes. In women, HIRI was associated with higher TAG and DAG levels, and lower odd-chain/even-chain TAG ratio. In men, no significant associations between HIRI and lipid classes were observed. Women exhibited less LIR and lower TAG levels compared with men, but showed more pronounced lipid profile deterioration with LIR progression.	DiOGenes^48^
Tissue-specific IR	Cross-sectional baseline metabolomics, post hoc stratification analysis; validation in independent cohort	634 participants (DiOGenes), non-T2D, BMI ≥27 kg/m², aged 18-65 y; 540 participants (Maastricht Study), non-T2D, BMI ≥27 kg/m², aged 40-65 y	No diet/treatment; plasma metabolites were analyzed under basal fasting conditions	In DiOGenes, both LIR and MIR exhibit a shared circulating metabolic profile characterized by higher (BC)AA (valine, isoleucine, oxo-isovaleric acid, alanine), lactate, and TAG, along with lower glycine levels. Liver IR also uniquely associates with reduced ketogenesis, reflected by lower ketone body (acetoacetate and 3-hydroxybutyrate) levels and increased ketogenic amino acids and derivatives (leucine, hydroxy-isobutyrate, tyrosine, proline, creatine, and N-acetyl). Validation in the Maastricht study results confirmed these findings for most metabolites, except for valine.	DiOGenes and Maastricht Study^39^

Abbreviations: AA, amino acid; ALT, alanine aminotransferase; AST, aspartate aminotransferase; ATP-III, Adult Treatment Panel III criteria for metabolic health in obesity; AUC, area under the curve; BCAA, branched-chain amino acid; BMI, body mass index; BW, body weight; CHD, coronary heart disease; CHO, carbohydrate; CRP, C-reactive protein; CVD, cardiovascular disease; DAG, diacylglycerol; FA, fatty acid; FPG, fasting plasma glucose; HbA1c, glycated hemoglobin; HDL, high-density lipoprotein; HFMM, high-fat mixed meal; HIRI, hepatic insulin resistance index; HOMA-IR, Homeostatic Model Assessment for Insulin Resistance; hsCRP, high-sensitivity C-reactive protein; IR, insulin resistance; LDL-C, low-density-lipoprotein cholesterol; LF, low-fat; LH, lean healthy; LIR, liver insulin resistance; LPC, lysophosphatidylcholine; MedDiet, Mediterranean diet; MetS, metabolic syndrome; MH, metabolically healthy; MHO, metabolically healthy obese; MIR, muscle insulin resistance; MISI, muscle insulin sensitivity index; MUFA, monounsaturated fatty acid; MUH, metabolically unhealthy; MUHO, metabolically unhealthy obese; NGT, normal glucose tolerance; NMR, nuclear magnetic resonance; NW, normal weight; OB, obese; OW, overweight; PRO, protein; PUFA, polyunsaturated fatty acid; RCT, randomized controlled trial; RE, remission; Rd, rate of glucose disposal; RRB, refined rye bread; RWB, refined wheat bread; SFA, saturated fatty acid; T2D, type 2 diabetes; TAG, triacylglycerol; TRL-TAG, triacylglycerol-rich lipoprotein-triacylglycerol; Vit. D, vitamin D; WRB, whole-meal rye bread.

### Tissue-Specific IR Is Associated With Changes in Branched-Chain Amino Acids, Ketone Bodies, and Lipid Metabolism

The whole-body IR metabolomic fingerprint is characterized by increased levels of branched-chain amino acids (BCAAs), aromatic AAs, glycolytic intermediates, lactate, TAG, long-chain fatty acids, and acylcarnitines, and decreased levels of ketone bodies, tricarboxylic acid cycle (TCA) intermediates, glycine, betaine, and lysophosphatidylcholines.^33^^,^^39^^,^^48^^,^^55^^,^^122^^,^^125–127^^,^^131^^,^^132^ Furthermore, discordant metabolic alterations can be observed with regard to tissue-specific IR metabotypes, as has been discussed previously (section entitled “A PN Strategy Focused on Tissue-Specific IR Metabotypes”). Namely, LIR adjusted for MISI is associated with increased serum fasting TAG, ketogenic AAs, and diacylglycerol (in women), and decreased KB levels, whereas MIR adjusted for HIRI is only associated with decreased lysophosphatidylcholines.^39^^,^^48^ Notably, these metabolic changes in LIR and MIR have been validated in 2 independent cohorts.^39^^,^^55^ Additionally, the adipose tissue IR index (ATIRI) correlates with increased BCAA, KB, lactate, and TAG levels, and decreased glycine levels.^32^^,^^33^^,^^39^^,^^48^^,^^55^  Table 5^39^^,^^48^ summarizes these tissue-specific IR changes in fasting metabolite levels. Recently, Gijbels et al^47^ characterized the postprandial metabolome of MIR and LIR. They found that LIR was, in particular, characterized by a more atherogenic postprandial lipoprotein profile—larger very-low-density lipoprotein (VLDL) and LDL and smaller HDL particles, and higher TAG, higher SFA, and lower PUFA levels—especially in women, as compared with MIR.

**Table 5.** Overview of Metabolites in Plasma Associated With Tissue-Specific IR Metabotypes and Whole-Body IR or T2D

Metabolite	Liver IR	Muscle IR	Adipose tissue IR
BCAAs	↑	↓	⬆
Valine	⇡	⇣	↑
Isoleucine	⬆	⬇	↑
Leucine (ketogenic)	↑	≈	↑
Oxo-isovaleric acid	⇡	⇣	↑
Hydroxy-isobutyrate	⇡	≈	↑
Amino acids (other)			
Alanine	↑	⬇	≈
Tyrosine	⬆	⇣	≈
Proline	⇡	≈	≈
Glycine	⇣	⇡	↓
Ketone bodies			
Acetoacetate	⬇	≈	↑
3-OH-butyrate	⬇	≈	↑
Glycolysis intermediates			
Lactate	↑	⬇	↑
Other metabolites			
Creatinine	⇡	⇡	≈
Creatine	⇡	≈	≈
Acetate	⇣	⇣	≈
Lipids and related			
Triacylglycerol	⬆	⬇	↑
Diglyceride	↑	≈	≈
Lysophosphatidylcholine	≈	↑	≈

The table’s main contents are based on the articles by Vogelzangs et al^39^ and Van der Kolk et al.^48^ Arrows indicate degree of change compared with population levels/control: ⬆, strong evidence for increase; ↑, moderate evidence for increase; ⇡, weak evidence for increase; ≈, equal/no change/no evidence; ⬇, strong evidence for decrease; ↓, moderate evidence for decrease; ⇣, weak evidence for decrease.

Abbreviations: BCAA, branched-chain amino acid; IR, insulin resistance; T2D, type 2 diabetes.

Others have also studied tissue-specific IR metabolite alterations, although have not adjusted for inter-tissue influences (ie, LIR adjusted for MIR and vice versa), which is essential for assessing independent associations.^39^^,^^48^^,^^126^^,^^132^ For example, Sunny et al^126^ observed that increased BCAA levels were more strongly associated with hepatic, adipose, and whole-body IR, while decreased BCAA levels were linked to muscle IR under fasting and insulin-stimulated conditions using a hyper-insulinemic-euglycemic clamp. This aligns with aforementioned and other studies (see Table S4).^39^^,^^48^^,^^127^ However, it should be noted that different methods for assessing hepatic and muscle IR were used than those previously introduced in the previously mentioned section.^19^^,^^41^^,^^42^

### Baseline Metabolome Profiles May Support Intervention Outcome Prediction

Within the population of individuals at risk of T2D, metabolic health status appears to be more strongly linked to observed changes in (postprandial) lipid metabolism, inflammatory status, and circulating metabolites, compared with BW status. For example, within the framework of the Spanish CORDIOPREV trial,^133^ Perez-Martinez et al^128^ compared the postprandial TAG and inflammatory response of, in total, 6 combinations of body size (normal-weight, overweight, obese) and metabolic health status (metabolically healthy vs unhealthy, with ≥2 MetS hallmarks) of participants with increased cardiovascular disease (CVD) risk.^128^ At baseline, 1002 participants underwent a high-fat mixed-meal test, with blood samples collected over a 4-hour postprandial period.^128^ Metabolically healthy individuals, regardless of obesity status, showed lower postprandial plasma TAG and large TAG-rich lipoproteins (chylomicrons and VLDL) levels.^128^ Conversely, metabolically unhealthy participants had lower postprandial hsCRP responses than their healthy counterparts, irrespective of body size.^128^ Additionally, a post hoc analysis was conducted in 190 participants with newly diagnosed T2D at baseline, comparing baseline metabolite levels between those who achieved remission and those who did not following a 5-year dietary intervention.^129^ At baseline, 12 endogenous metabolites, including rare fatty acids, bile acid metabolites, and creatine, differed between groups.^129^ A regression probability score integrating these metabolites with clinical variables (sex, age, BMI, HDL, TAG, and statin therapy) significantly enhanced T2D remission prediction compared with the use of clinical variables alone.^129^

Consistent with the aforementioned findings and expanding upon them, Urpi-Sarda et al^130^ characterized the urinary metabolomic fingerprint of participants enrolled in the PREDIMED (Prevención con Dieta Mediterránea) trial,^134^^,^^135^ which was a 5-year RCT that aimed to assess effects of a MedDiet on CVD prevention. This cross-sectional study revealed 33 significantly different metabolites when comparing individuals with T2D to those without (non-T2D), with all participants presenting at least 3 CVD risk factors.^130^ The T2D profile indicated alterations in carbohydrate and AA metabolism, with increased excretion of glucogenic Aas, such as alanine and phenylalanine (including derived metabolites), and decreased excretion of glutamine and histidine, potentially indicative of protein degradation and their utilization in gluconeogenesis (see Table S4).^130^ Subsequently, a k-means cluster algorithm was used on the aforementioned metabolites to identify discordant metabolome profiles among the groups, resulting in a further subdivision of both T2D and non-T2D groups.^130^ This analysis revealed that the T2D cluster with the highest plasma glucose levels also had the highest levels of acetoacetate, p-cresol, phenylalanine, and phenylacetylglutamine (PAG)—suggesting a link with decreased metabolic control, as observed previously.^96^^,^^130^ Interestingly, as the authors also indicate, some of these metabolites (ie, p-cresol and PAG) are proteolytic products of the gut microbiome; thus, dietary strategies that improve gut microbial composition and saccharolytic formation capacity in the more metabolically compromised clusters may be an interesting target for dietary intervention.^130^

As illustrated here and in preceding paragraphs, (microbial) metabolites, including BCAAs, AAs, TMAO, and p-cresol, are increasingly linked to metabolic status and may predict the onset of disease at an early stage.^96^^,^^122^ Despite the comprehensive overview provided of distinct metabolite profiles in tissue-specific IR and T2D, there is currently no evidence from prospective studies demonstrating successful improvement in metabolic health using metabolome-based dietary modulations.

### Prediction models in PN

As is evident from the previously discussed studies, the field of PN is complex, wherein multiple factors are at play that may also display cross-talk with each other. To overcome this complexity, recent advancements incorporate either Advanced Statistics or AI methodologies, such as ML, to identify and cluster distinct phenotypes or predict response/nonresponse to dietary interventions. Importantly, these methodologies can be untargeted or more targeted, incorporating specific subsets of variables that are known to be diet-sensitive.^136–138^ Our search and selection revealed 8 studies that incorporated prediction models or clustering methodologies in their study design. (See Table 7^16–18^^,^^139–142^ for an overview of included studies in this section.) In addition, 6 relevant studies were included from their reference lists via handsearching.

**Table 7.** Overview of Dietary Interventions That Incorporated Prediction Model–Based Stratification

Metabotyping/stratification	Study design	Participants	Diet/treatment	Outcome	Reference
PPGR	ML algorithm, incorporating 1-wk CGM monitoring, dietary intake, test meals, blood data, anthropometrics, PA, behavior, and gut microbiome	800 participants healthy and prediabetic (Discovery cohort), independent validation cohort (*n* = 100) and RCT (*n* = 26)	Validated in independent cohort and 1-wk randomized crossover design. “Prediction” (algorithm) arm, hidden PPGRs vs “Expert” (dietitian) arm, with visible PPGRs (CGM) from the profiling week to select “good” vs “bad” diet.	In the predictor arm, 10/12 participants had significantly higher PPGRs on the “bad” diet compared with the “good” diet. The predictor arm showed comparable success to the expert arm, where 8 out of 14 participants experienced significantly lower PPGRs on the “good” diet vs the “bad” diet. Both approaches were effective in reducing PPGRs through personalized nutrition. However, the predictor-based approach is more broadly applicable as it can predict PPGRs for any meal, unlike the expert-based approach, requiring CGM data.	Zeevi et al^16^
PPGR/PPT	6-mo, parallel-design RCT, with 6-mo follow-up	225 prediabetic (FPG 5.6-6.9 mmol/L and HbA1c 39-48 mmol/mol) participants, aged 18-65 y; 177 included in follow-up	Isocaloric PPT diet based on PPGR algorithm,^16^ incorporating clinical and microbiome features vs MedDiet control. The PPT diet was lower in CHO content (20.4E%), compared with the MedDiet (45-65E%).	At 6-mo, improvements in primary outcomes, mean glucose time above 7.8 mmol/L (CGM) and HbA1c, were significantly better in PPT vs the MedDiet: respectively, -1.3 to -0.3 h/d and -1.7 to -0.9 mmol/mol. At 12-mo follow-up, significant between-group differences were maintained in PPT vs the MedDiet: respectively, -1.4 to -0.2 h/d and -1.2 to 0.4 mmol/mol. Secondary outcomes: no significant between group difference in 2-h change in glucose levels (CGM) were found following OGTT. Significantly different blood lipid profile improvements (TAG, HDL, and TC/HDL ratio) were observed comparing PPT vs the MedDiet. No significant between-group differences were found for FPG, FI, and HOMA-IR. No other measures of IR were reported.	Ben-Yacov et al^18^
PPGR/PPLR/PPIR	ML algorithm incorporating baseline characteristics, biochemistry, anthropometry, genetics, microbiome, diet, and meal context	1002 twins and unrelated healthy participants, aged 18-65 y in Discovery cohort; independent validation cohort (*n* = 100)	PPGR/PPLR/PPIR response prediction model was trained following 7 standardized test meals, in duplicate (clinic and at-home). Glucose data were obtained by CGM, C-peptide from serum and TAG from DBS assays.	The ML model predicted individual responses in the validation cohort with reasonable accuracy (correlation predicted vs measured), particularly for glucose. Specifically, 0.47 vs. 0.42 for TAG_6h-rise_, 0.77 vs 0.75 for glucose_iAUC0-2h_ and 0.30 vs 0.14 for C-peptide_1h-rise_, respectively. Results indicate that meal composition, individual variability (eg, microbiome, genetics), and modifiable factors such as meal timing significantly and differentially affect postprandial glycemia and lipidemia.	PREDICT 1^17^
PPGR/PPT	6-mo, parallel-design RCT, post hoc stratification analysis	200 prediabetic (FPG 5.6-6.9 mmol/L and HbA1c 39-48 mmol/mol) participants, aged 18-65 y	Isocaloric PPT diet based on PPGR algorithm,^16^ incorporating clinical and microbiome features vs MedDiet control.	The PPT arm reduced CHO intake (−17.9% to −15.6% kcal) and dietary fiber (−2.4 to −1.0 g/1000 kcal), while the MedDiet arm slightly increased carbohydrate intake (1.0% to 2.5% kcal) and dietary fiber (2.9 to 4.0 g/1000 kcal). Total dietary fats and their subtypes (SFAs, MUFAs, PUFAs) increased in the PPT arm and decreased in the MedDiet arm. PRO intake slightly increased in both arms. The PPT diet induced significant changes in gut microbiome composition, including increased alpha-diversity, while no significant changes were observed in the MedDiet arm. Associations between dietary changes and microbiome species-level changes were identified, with 9 microbial species (eg, *Bacteroidales*, *Lachnospiraceae*, *Oscillospirales*), partially mediating the effects of dietary changes on clinical outcomes such as HbA1c, HDL-C, and TAG.	Ben-Yacov et al^139^
PPGR/PPLR/PPIR and gut microbiome	Repeated baseline meal challenge test, combined with post hoc analysis	1098 participants (both twins and unrelated), aged 18-65 y; no reported body-composition restriction	7 Standardized test meals, in duplicate (clinic and at-home). Glucose data were obtained by CGM, C-peptide from serum and TAG from DBS assays.	Participants were extensively profiled with regard to anthropometrics, blood biomarkers, habitual diet, CGM, and stool metagenomics. The presence of *Prevotella copri* and *Blastocystis* species, uncommon in individuals with OB, was strongly associated with improved postprandial glucose tolerance, C-peptide (0–2 h iAUC), TAG (0–6 h iAUC), and reduced VAT mass. A synergistic effect was observed with both species present: VAT mass was 17.3% lower compared with those positive for only 1 species and 23.3% lower compared with individuals lacking both.	PREDICT 1^140^
Gut microbiome and genetics	4-mo, parallel-design RCT, post hoc stratification analysis	190 participants, BMI 25–40 kg/m², aged 18-67 y	Hypocaloric (-30%) MHP (40E% CHO, 30% PRO, 30% fats) vs LF (60E% CHO, 18% PRO, 22% fat).	Baseline gut microbiome DNA sequencing and participant DNA genotyping were used to construct respective microbiota (diet-specific genera, families, and species) and genetic sub-scores (6 and 7 SNPs for MHP and LF, respectively). The subsequently constructed BMI loss prediction model accurately predicted the most suitable diet for WL based on both sub-scores. The microbiota sub-score enabled selection of the optimal diet in 72% of women and 84% of men, while the genetic sub-score did so in 84% of women and 73% of men.	Cuevas-Sierra et al^141^
Low-TMAO vs high-TMAO producer	30-d parallel-design, post hoc stratification analysis	56 participants, of whom 23 were vegetarian and 33 omnivorous	Carnitine fumarate supplementation (500 mg/d). Pre- and post-OCCT (1500 mg of carnitine tablets).	Following carnitine supplementation, the proportion of omnivorous subjects with potentially harmful plasma TMAO levels (>10 μM) increased from 3% to 21%, while none were high TMAO producers among vegetarians. Gut microbial analysis, identified 39 OTUs (including *Emergencia timonensis* and *Ihubacter massiliensis*), which highly correlated with TMAO production. A random-forest classifier was able to predict TMAO producer phenotypes with an AUROC of 0.81, validated by an external cohort (AUROC = 0.80). The presence of *E timonensis* and *I massiliensis* accounted for 43% of high TMAO producers, with 97% specificity.	Wu et al^142^

Abbreviations: AUROC, area under the receiver operating characteristic curve; BMI, body mass index; CGM, continuous glucose monitoring; CHO, carbohydrate; DBS, dried blood spot; E%, percentage of energy; FI, fasting insulin; FPG, fasting plasma glucose; HbA1c, glycated hemoglobin; HDL, high-density lipoprotein; HDL-C, high-density-lipoprotein cholesterol; HOMA-IR, Homeostatic Model Assessment for Insulin Resistance; iAUC, incremental area under the curve; LF, low-fat diet; MedDiet, Mediterranean diet; MHP, moderately high-protein diet; ML, machine learning; MUFA, monounsaturated fatty acid; OB, obesity; OCCT, oral carnitine challenge test; OGTT, oral-glucose-tolerance test; PA, physical activity; PPGR, postprandial glycemic response; PPIR, postprandial insulinemic response; PPLR, postprandial lipidemic response; PPT, postprandial targeting diet; PRO, protein; PUFA, polyunsaturated fatty acid; RCT, randomized controlled trial; SFA, saturated fatty acid; SNP, single nucleotide polymorphism; TAG, triacylglycerol; TC, total cholesterol; TMAO, trimethylamine N-oxide; VAT, visceral adipose tissue; WL, weight loss.

For example, ML algorithms are increasingly being used to predict improvements in markers of cardiometabolic health upon dietary modulation, incorporating (baseline) data on diet–microbiome–host interactions.^16–18^^,^^139^^,^^140^^,^^143^ Most notably, Zeevi et al^16^ conducted one of the most highly referenced papers in this field, being the first to show feasibility of personalized postprandial glucose response (PPGR) predictions based on an ML algorithm (*r* = 0.68), that included data on microbiota composition, metabolism, and lifestyle. This algorithm was validated in an independent cohort (*n* = 100, *R* = 0.70), and a subsequent small-scale RCT of only 1 week, comparing dietary advice between the algorithm and expert dietitians, and showed that it was able to significantly and comparably lower PPGRs and improve gut microbial composition.^16^ Building upon the previously developed algorithm that was able to predict PPGRs, Ben-Yacov et al^18^ were able to demonstrate the beneficial cardiometabolic effects of a personalized postprandial-targeting (PPT) diet, which closely resembled a low-CHO diet (20.4 E%), compared with a MedDiet control in prediabetic^18^ and newly diagnosed diabetic^144^ individuals. In a subsequent post hoc analysis, they were able to demonstrate that the PPT diet induced more prominent changes in the gut microbial composition (alpha-diversity) compared with the MedDiet control.^139^ Interestingly, they were able to pinpoint 9 microbial species (eg, from the orders Bacteroidales, Lachnospiraceae, and Oscillospirales) that partially mediated associations between improvements clinical outcomes (HbA1c, HDL cholesterol, and TAG) and induced dietary changes.^139^ In another post hoc multi-omics analysis of the same intervention, Shoer et al^145^ found that 166 of the 2803 measured features, including fecal and oral microbiota composition and pathways, serum metabolites, and cytokines, significantly changed in response to the PPT diet compared with the MedDiet control. This analysis also re-confirmed the previously found mediating effect of changes in the gut microbiota composition on diet-induced improvements in glycemic, metabolic, and immune function markers, wherein microbiome change explained 12% of variance in serum metabolites.^145^

Within the larger PREDICT trial,^17^ a dietary intervention study exploring the interactions between diet, genetics, gut microbiome, and cardiometabolic health, several noteworthy findings were replicated across independent cohorts. Berry et al^17^ conducted a comprehensive study assessing personalized postprandial responses across 1002 healthy twins and unrelated participants (aged 18–65 years). The participants received 7 standardized test meals in duplicate both in clinical and home settings, whereafter a prediction model was developed. This ML model integrated various input variables, including baseline characteristics, clinical biochemistry (ie, glucose, C-peptide, and TAG), anthropometry, genetics, gut microbiome, habitual diet, and meal context and composition.^17^ The model was subsequently validated in an independent cohort, yielding similar correlations (except for C-peptide) when comparing predicted and observed values—namely, 0.47 vs 0.42 for 6-hour rise in TAG, 0.77 vs 0.75 for 0–2-hour glucose iAUC, and 0.30 vs 0.14 for 1-hour rise in C-peptide, respectively.^17^ Interestingly, Berry et al^17^ discerned the impact of meal macronutrient composition and person-specific factors on predictions. Notably, while meal composition had a smaller influence compared with the gut microbiome on postprandial lipemia (3.6% vs 7.1% of variance, respectively), the reverse was observed for postprandial glycemia (15.4% vs 6.0%). Moreover, genetics exhibited a more modest influence on predictions (9.5% for glucose, 0.8% for TAG, and 0.2% for C-peptide). In addition, within the PREDICT trial, Asnicar et al^140^ consistently found a set of microbial species to be strongly linked to dietary indices, indicators of obesity and cardiometabolic health, fasting circulating metabolites, and postprandial response. For instance, the presence of *Prevotella copri* and *Blastocystis* species, which is regarded as uncommon in individuals with obesity, was found to be a strong indicator of more favorable postprandial glucose tolerance, insulin, C-peptide (all 0–2-h iAUC), TAG (0–6-h iAUC), and visceral fat mass.^140^ Interestingly, a potential synergistic effect of the simultaneous presence of both species could be observed, as, for instance, average visceral fat mass was 17.3% lower in individuals positive for both species compared with those positive for only one, whereas this difference was even larger (23.3%) compared with those individuals lacking both species.^140^

In addition to the aforementioned landmark studies conducted by Zeevi et al^16^ and Berry et al^17^ more simplified prediction models or clustering strategies are also used; however, these are usually not validated in independent cohorts or subsequent RCTs. For example, in a study by Cuevas-Sierra et al,^141^ participants were randomized to receive either a hypocaloric, moderately high-protein (MHP; 40% CHO, 30% protein, 30% fat) or LF (60% CHO, 18% protein, 22% fat) diet for 4 months. Post hoc, a BMI loss prediction model accurately predicted the most suitable diet for weight loss based on gut microbiota (diet-specific genera, families, and species) and genetic subscores (6 and 7 SNPs for MHP and LF, respectively).^141^ The microbiota subscore enabled selection of the optimal diet in 72% of women and 84% of men, while the genetic subscore did so in 84% of women and 73% of men.^141^ In another post hoc analysis, Seethaler et al^143^ were able to predict improvements in intestinal barrier function following adherence to a MedDiet for 3 months, based on baseline fecal SCFA data using an ML algorithm.^143^ Conversely, instead of predictions regarding interindividual differences in production capacity of beneficial microbial metabolites (eg, SCFAs), similar diet–response predictions regarding metabolites detrimental to host health (eg, TMAO) could be made.^142^

Similar initiatives have also been undertaken within the domain of T2D prevention by means of physical exercise. For instance, Liu et al^146^ demonstrated that exercise training in a discovery cohort of individuals with prediabetes resulted in high interpersonal variability in response with regard to improvements in FPG, FI, and HOMA-IR. In responders, induced changes in microbial composition correlated with metabolic improvements, resulted in enhanced SCFA production capacity, and increased catabolism of BCAAs. Subsequently, a developed ML algorithm, incorporating differentially abundant features between responders and nonresponders (ie, baseline microbial composition and metabolites) was able to accurately predict glycemic response to exercise in a validation cohort (*n* = 30).^146^

The aforementioned overview demonstrates that, especially within the field of PN, the use of ML and advanced modeling techniques has propelled substantial developments. However, to date and to the best of our knowledge, apart from studies by Zeevi et al,^16^ Ben-Yacov et al,^18^ and Berry et al,^17^ no other studies have validated their findings in independent cohorts or subsequent prospective studies, which remains a crucial step before implementing these strategies in clinical practice. Furthermore, a clear understanding of mechanisms underlying the differential glycemic responses and how these relate to cardiometabolic outcomes may be required to be able to implement PN strategies into the healthcare setting or into dietary guidelines.^96^^,^^140^^,^^147^

## PRECISION NUTRITION IN PREVENTION OF T2D AND CARDIOMETABOLIC DISEASE: CURRENT AND FUTURE PERSPECTIVES

The present scoping review aimed to summarize advancements in the field of PN over the past decade, focusing on heterogeneity in response (ie, cardiometabolic health improvement) to dietary interventions in individuals with overweight and obesity. Intervention personalization can be based on metabotypes, genotype or microbial phenotype, either as a stand-alone intervention or combined with AI/ML techniques. Our PubMed search identified 706 publications from 1978 to 2024, with 526 published in the last 10 years and 298 in the last 5 years, underscoring the growing interest and effort in developing effective PN strategies for T2D prevention.

Many post hoc analyses underscored the potential of macronutrient modulations to effectively manage (postprandial) glucose metabolism, body composition, and weight, yet limited prospective studies have confirmed these findings to date. A notable exception is the PERSON study by Trouwborst et al,^19^ which is the first prospective trial demonstrating that eucaloric macronutrient modulations within dietary guidelines, tailored to tissue-specific IR phenotypes, significantly improves cardiometabolic health, regardless of changes in BW. Specifically, an LF, high-protein, and high-fiber diet proved to be more effective for the MIR metabotype, while a higher-fat, high-monounsaturated-fat diet was more beneficial for the LIR metabotype.^19^ In line, a recent study by Wang et al^148^ indicates that mechanism-based dietary modulations tailored to specific etiologies are most effective in preserving metabolic health, showing that diets designed to prevent hyperinsulinemia or inflammation were more effective than general healthy diets in preventing metabolic disease.^148^ The authors suggest focusing on dietary components that mechanistically influence disease etiology when designing nutritional interventions, of which the PERSON study can be regarded as a notable example.^148^

Within the scope of nutrigenetics, earlier studies in the past decade have focused on gene–diet interactions associated with isolated SNPs. However, there remains a notable gap in evidence supporting highly effective dietary recommendations based on multiple SNPs or genome-wide analyses, particularly when combined with other biomarkers.^88^ For example, prospective studies that incorporated genotyping, such as the PREVENTOMICS,^83^^,^^84^ Food4Me,^62^^,^^68^^,^^85^^,^^87^ Women’s Health Initiative,^77^ and Personalized Nutrition Study (POINTS),^80^ have not provided evidence supporting the added value of incorporating genotype data in an intervention design. As previously discussed, there are still some prospective studies based solely on genotype–diet interactions that demonstrate enhanced weight loss^73^^,^^74^^,^^79^ and improved body composition^74^ and inflammatory profiles.^76^ However, these prospective studies in the field of genotype-based PN remain scarce, largely due to the complexity of their design and execution. The framework proposed by Grimaldi et al,^149^ as part of the Food4Me project, highlights important implications for evaluating scientific evidence on gene–diet interactions and for designing future prospective studies in the field of PN. It establishes minimal guidelines regarding study design, quality, and the biological plausibility of findings, providing a structured approach to advance research in this area.^149^ Hereby, it aims to ensure that nutrigenetic advice is grounded in scientifically robust interpretation, providing transparent and reliable recommendations while protecting the public from misuse.^149^

The herein identified studies demonstrate that microbial composition, diet, and circulating metabolites are closely linked, wherein baseline microbial composition (responders vs nonresponders) may be one of the determinants of the impact of diet on the hosts’ metabotype.^17^^,^^49^^,^^95^^,^^96^^,^^109^^,^^112^^,^^113^^,^^140^ This is exemplified by the intricate balance between beneficial saccharolytic fermentation (producing SCFAs) and detrimental proteolytic fermentation (producing BCAAs, AAs, BCFAs, TMAO, p-cresol), in which disturbances can already be observed long before onset of disease and distinct phenotypes towards cardiometabolic disease may be observed.^39^^,^^48^^,^^96^^,^^122^ From this perspective a whole-diet approach that focuses on increasing colonic saccharolytic fermentation and decreasing detrimental proteolytic metabolites is warranted, which may significantly impact metabolic health and weight management.^47^^,^^96^^,^^122^

The pioneering studies conducted by Zeevi et al,^16^ later followed by Berry et al,^17^ first used an ML approach for the prediction of postprandial glycemic response and diet–microbiome–disease associations. This integration of big data, ML, multi-omics, and advanced modeling techniques has driven substantial progress in developing evidence-based PN strategies that influence diet–microbiome–host interactions.^96^^,^^140^^,^^147^ With the exception of the above-mentioned studies, very few have managed to validate their findings in independent cohorts or subsequent prospective studies^16–19^^,^^139^ (see also Table 1 and Table 7 for a detailed overview of these seminal prospective studies in PN). When integrating such novel technologies into intervention design, future studies should aim to adopt targeted, evidence-based strategies that provide a clear understanding of the underlying mechanisms driving improvements in glycemic control and overall cardiometabolic health. Importantly, researchers should avoid relying on black-box algorithms—that is, complex models (often ML-based) whose internal decision-making processes are not transparent or interpretable—particularly when these are used to guide dietary recommendations.^96^^,^^140^^,^^147^

In dietary intervention studies, the inclusion of multi-omics measurements often generates large datasets, while sample sizes typically remain small (<200 participants), presenting both opportunities and challenges. Especially here, ML excels at integrating diverse datasets and uncovering relationships between diet, ’omics data, and health outcomes, making it highly effective for identifying (subgroups of) responders or development of predictive models. However, the small sample sizes increase the risk of overfitting, compromising the generalizability of findings. The sometimes “black box” nature of ML algorithms can also obscure biological mechanisms, complicating interpretation and application. Finally, validation in larger, independent cohorts is frequently challenging, further limiting the translational potential of many findings.

Furthermore, some post hoc analyses of dietary intervention studies could be criticized for “cherry-picking” outcomes, which—while insightful for understanding response variability—may lack robustness. More systematic approaches that compare minimally clinically important differences with standard deviations of individual response may better account for intrinsic inter- and intraindividual variability.^150^ Such approaches allow to differentiate true variability in response from random measurement error or within-subject variation, therefore being more meaningful for the identification of response determinants in PN.^150^

Our review only briefly addresses behavioral and psychological aspects of food intake and adherence to an intervention and their impact on metabolic health.^87^ Factors such as the intensity of intervention delivery—whether face-to-face, online, or via leaflets—play a significant role in dietary adherence, intervention outcomes, and (non-)response.^87^^,^^151^ Moreover, complex psychological factors, including gut–brain axis regulation of food intake, as evidenced in studies on bariatric surgery, are critical determinants of compliance and the obesity-related health effects of dietary interventions.^152–154^ These aspects certainly have to be taken into account when evaluating the effectiveness of PN and warrant further exploration in the context of PN. Furthermore, there is growing focus on how ethnicity or ancestry influences cardiometabolic responses to diet, resulting in variation in metabotypes, genotypes, and gut microbial phenotypes.^155–161^ Black individuals, for instance, show increased vulnerability to T2D but paradoxically have lower TAG and higher HDL-cholesterol levels.^158^ Evidence suggests that this can be modulated by degree of European ancestry, which is associated with decreased T2D risk, yet concomitant increased TAG and decreased HDL levels.^158^ Although previously attributed to socioeconomic status (SES), studies correcting for SES still show significant interactions between diet and health outcomes, indicating that ethnic ancestry may have a more substantial impact on T2D incidence than SES.^157^^,^^158^ Taking the aforementioned constraints into consideration is crucial for generating replicable results and facilitating the translation of scientific study findings into evidence-based PN interventions suitable for implementation into daily clinical practice.

A critical focus of PN should be on understanding the modifiable factors driving nonresponsiveness, which will enable tailored interventions to convert nonresponders into responders. Nonresponders may exhibit milder degrees of IR or cardiometabolic disturbances^21^^,^^32–34^ or may require alternative nutritional or lifestyle interventions,^4^^,^^19^ meaning they could still benefit from more tailored and targeted nutritional strategies. For instance, a previously mentioned combined fiber intervention revealed that healthy lean participants exhibited improved glucose control and metabolic parameters, whereas individuals with overweight, obesity, and prediabetes did not.^116^ This underscores that nonresponders may not always be those with milder cardiometabolic disturbances, yet may require entirely different PN interventions to elicit a response. More importantly, PN should not aim to only understand isolated highly responsive metabotypes, but should strive for a clear mechanistic understanding of all optimal treatments for metabotypes to elicit the same positive outcomes. For instance, the extensively studied PERSON trial^19^ identified 2 highly responsive metabotypes to specific macronutrient modulations. However, these metabotypes accounted for only 27.6% of the at-risk population, leaving unanswered the critical question of whether similarly optimal treatments can be defined for the remaining 72.4%.

Clustering methodologies offer a holistic approach by characterizing diverse phenotypes, including less-responsive individuals, rather than prioritizing highly responsive subtypes alone, which may identify the optimal intervention for all subtypes. Clustering incorporating multiple, accurate clinical and metabolic features of the prediabetic state, as has already been implemented in other fields, may more closely align with a participant’s clinical phenotype and accurately predict response to an intervention.^136^ This allows for initial detailed characterization of phenotypes identified in large datasets incorporating many body-composition features and biomarkers. Subsequent development of a classification algorithm based on these phenotypes’ characteristics, which only requires a few simplified measurements with a high phenotype predictive value, may allow to bridge the gap between clinical studies and wider application in daily practice. Ideally, these measurements can be performed in an at-home setting utilizing web-based dashboards, easy-to-use test kits, and/or wearables that may be able to replace invasive clinical phenotyping in the future to identify and select optimal dietary treatment.^30^^,^^38^ These inclusive strategies ensure that PN frameworks address the needs of all at-risk individuals, enhancing their applicability, scalability, and effectiveness. Furthermore, these wearables and self-monitoring devices may represent an intervention in themselves through stimulating healthy behavior and dietary adherence.

## CONCLUSION

It remains evident that a more personalized dietary approach can be a promising avenue for the broader population of at-risk individuals, warranting more inclusive classification strategies. A clear mechanistic understanding of response and nonresponse to dietary interventions in terms of cardiometabolic outcomes remains especially crucial for developing PN strategies that effectively prevent the onset of T2D.

## Supplementary Material

nuaf088_Supplementary_Data

## Data Availability

No new data were generated or analyzed in this study. All data supporting the findings of this review are derived from published sources, as cited in the manuscript.
