# Supplementary material for: Metabolic Phenotypes, Genotypes, and Gut Microbiome Signatures in Obesity: Implications for Precision Nutrition Strategies in Type 2 Diabetes Prevention
Source: Nutr Rev. 2025 Dec 9;84(1):158–88. doi: 10.1093/nutrit/nuaf088 (PMC12696375; doi:10.1093/nutrit/nuaf088)
Supplement: nuaf088_Supplementary_Data [file nuaf088_supplementary_data.docx]

Supplementary material

Table of Contents

[Table S1: Search string used in the semi-systematic search for the present work. 1](#_Toc201152055)

[Table S2: In- & exclusion criteria 2](#_Toc201152056)

[Figure S1: PRISMA diagram of the present work. 4](#_Toc201152057)

[Table S3: Extended overview of Metabolites in plasma and/or urine associated with tissue-specific IR Metabotypes and whole-body IR or T2DM.. 5](#_Toc201152058)

## Table S1: Search string used in the semi-systematic search for the present work.

| For the present scoping review, a semi-systematic PubMed search was conducted to find relevant literature. Our search strategy combined the use of the following search terms and MeSH, supplemented by using the Boolean operators ‘’OR’’ and ‘’AND’’, specifically: |
| --- |
| (precision[tiab] OR precise[tiab] OR personalized[tiab] OR personalized[tiab] OR targeted[tiab] OR stratified[tiab]) AND (‘’precision nutrition’’[tw] OR ‘’personalized nutrition’’[tw] OR ‘’personalized nutrition’’[tw] OR ‘’metabolic phenotype*’’[tw] OR metabolic[tw] OR phenotype*[tw] OR sub-phenotype*[tw] OR metabotyping[tw] OR metabotype*[tw] OR genotype*[tw] OR microbiome[tw] OR "Precision Medicine"[Mesh] OR "Phenotype"[Mesh]) AND (overweight[tw] OR obesity[tw] OR ‘’metabolic syndrome’’[tw] OR diabetes[tw] OR ‘’type 2’’[tw] OR ‘’diabetes mellitus’’[tw] OR ‘’insulin resistance’’[tw] OR ’’insulin’’[tw] OR ‘’glucose homeostasis’’[tw] OR ‘’tissue-specific insulin resistance’’[tw] OR ‘’cardiovascular disease*[tw] OR ‘’cardiometabolic health’’[tw] OR "Diabetes Mellitus, Type 2"[Mesh] OR "Insulin Resistance/physiology"[Mesh] OR "Insulin"[Mesh] OR "Metabolic Syndrome"[Mesh] OR "Overweight"[Mesh] OR "Obesity"[Mesh] OR "Cardiovascular Diseases/prevention and control"[Mesh]) AND (human*[tw] OR intervention[tw] OR ‘’dietary intervention’’[tw] OR trial[tw] OR random*[tw] ‘’middle aged’’[tw] OR aged [tw] OR older[tw] OR "Humans"[Mesh] OR ‘’Diet Therapy’’ [Mesh] OR "Adult"[Mesh] OR "Middle Aged"[Mesh] OR "Aged"[Mesh]). |

## Table S2: In- & exclusion criteria

| INCLUSION | EXCLUSION |
| --- | --- |
| POPULATION | |
| - Human adults, aged ≥18 years, - Healthy, normo-glycemic participants, - Participants at risk of or with overweight/obesity - Participants at risk of overweight/obesity and/or Metabolic Syndrome or associated cardiometabolic disorders/diseases, such as;   - Pre-diabetes, Insulin Resistance, Glucose Intolerance, Hyperglycemia,   - Metabolic-Associated Fatty Liver Disease / Non-Alcoholic Fatty Liver Disease   - Hypercholesterolemia, Hyperlipidemia   - (Chronic) Low-Grade Inflammation   - Cardiovascular disease, incl. Hypertension | - Studies (solely) in children (i.e., at inclusion ≤18 years of age) - Studies solely with participants already diagnosed with Type 2 Diabetes Mellitus - Participants diagnosed with disorders/diseases other than those mentioned in inclusion criteria such as;   - All types of cancer,   - Auto-immune disease (e.g., type 1 diabetes)   - Gastrointestinal disorders/disease,   - Psychological disorders/disease |
| INTERVENTION / TREATMENT CONDITION | |
| - Participants should have been stratified (either prospective or post-hoc) by group or on at the individual level based on their;   - (degree of) Glucose homeostatic disturbances   - Anthropometrics / adiposity   - Genetics,   - Microbiome composition/functionality,   - Metabolome / Lipidome,   - Postprandial glycemic response   - Inflammatory status,   - Ethnicity,   - Or a combination thereof. | - Any other basis for stratification other than those mentioned in inclusion criteria. |
| COMPARATOR / CONTROL CONDITION | |
| - Precision- or Personalized dietary interventions, tailored to one (or multiple) of the stratification criteria mentioned in the intervention condition, that compare (to):   - Population-based general/conventional dietary guidelines   - Widely accepted/recognized healthy diets (e.g., New Nordic- or Mediterranean- diet)   - (Hypothesized) Optimal- vs. Suboptimal diets   - Varying degrees of Precision- or Personalized treatment intensities   - Studies without a control group (e.g., observational or n-of-1 studies) | - Studies including non-dietary interventions/treatment (e.g., surgery, physical activity, medication, psychological care) - Other types of studies than those mentioned in inclusion criteria. |
| STUDY TYPE | |
| - Clinical- and Randomized Controlled Trials (RTCs), incl. studies employing post-hoc stratification of these (R)CTs - Observational studies - Publication date between years 2014-2024 - Publications written in English | - Studies focusing on data from animal and/or in vitro models (Hybrid studies with human data are allowed). - Reviews and meta-analysis - Other types of studies than those mentioned in inclusion criteria. |
| OUTCOME PARAMETERS | |
| - The main outcome parameter should be prevention or management of the cardiometabolic disorders/diseases mentioned under ‘’population’’. | - Other types of studies than those mentioned in inclusion criteria. |

## Figure S1: PRISMA diagram of the present work.

## Table S3: Extended overview of Metabolites in plasma and/or urine associated with tissue-specific IR Metabotypes and whole-body IR or T2DM. The table main contents are based on the article by Vogelzangs et al.^39^, and supplemented with results from mentioned studies within this section.

| Metabolite | Liver IR | Muscle IR | Adipose Tissue IR | Whole-body IR / T2DM | Reference |
| --- | --- | --- | --- | --- | --- |
| BCAA & derivatives | ⬆︎ | ⇣ | ⬆︎ | ↑^*^ | ^39, 126, 127, 132^ |
| - *Valine* | ↑ | ↑↓ | ↑ | ↑^*^ | ^39, 126^ |
| - *Isoleucine* | ↑ | ↓ | ↑ | ↑^*^ | ^39, 126, 127^ |
| - *Leucine (ketogenic)* | ↑ | ↓ | ↑ | ↑^*^ | ^39, 126, 127^ |
| Amino acids & derivatives |  |  |  |  |  |
| - *Alanine* | ⬆︎^*^ | ↑↓ | ↑ | ↑^U*^ | ^39, 126, 127^ |
| - *Tyrosine* | ↑^*^ | ⇣^*^ | ↑ | ↑ | ^39, 126^ |
| - *Proline* | ↑^*^ | ⇣ | ⇡ | ⇡ | ^39, 126^ |
| - *Glycine* | ↓? | ↓? | ↓ | ↓? | ^39^ |
| - *Phenylalanine* | ↑ | ↓ | ⇡ | ↑^U*^ | ^126, 130^ |
| - *Creatine* | ⇡? |  |  | ↑* | ^39, 129^ |
| - *Acetyl carnitine* | ↑ | ⇣ | ↑ | ↑^(↓in U)^ | ^126, 130^ |
| Ketone bodies |  |  |  |  |  |
| - *Acetoacetate* | ↓^*^ | ≈ | ↑ | ↓>↑^U#^ | ^39, 130^ |
| - *3-OH-butyrate* | ↓^*^ | ≈ | ↑ | ↑^U^ | ^39, 130^ |
| Carbohydrate metabolism & related |  |  |  |  |  |
| - *Lactate* | ↑ | ↑ | ↑ | ↑^*^ | ^39, 130^ |
| Gut microbiome |  |  |  |  |  |
| - *TMAO* |  |  |  | ↑^U^ | ^130^ |
| - *TMA* |  |  |  | ↓^U^ | ^130^ |
| - *p-Cresol* |  |  |  | ↓>↑^U#^ | ^39, 130^ |
| Lipids |  |  |  |  |  |
| - *Triacylglycerol* | ↑ | ↑ | ↑ | ↑? | ^39^ |

Arrow indicates degree of change compared to population levels / control. ⬆︎, High increase; ↑, increase; ⇡, slight increase; ≈, equal/no change; ⬇, high decrease; ↓, decrease; ⇣, slight decrease; ↑↓, indicates conflicting evidence regarding increased/decreased levels; U, also identified in Urine. An asterisk (*) indicates that these metabolite changes were observed in multiple datasets/studies. # indicates an increase following and increase in FPG.
